# Genome-wide association study of COVID-19 severity among the Chinese population

**DOI:** 10.1038/s41421-021-00318-6

**Published:** 2021-08-31

**Authors:** Yuanfeng Li, Yuehua Ke, Xinyi Xia, Yahui Wang, Fanjun Cheng, Xinyi Liu, Xin Jin, Boan Li, Chengyong Xie, Siyang Liu, Weijun Chen, Chenning Yang, Yuguang Niu, Ruizhong Jia, Yong Chen, Xiong Liu, Zhihua Wang, Fang Zheng, Yan Jin, Zhen Li, Ning Yang, Pengbo Cao, Hongxia Chen, Jie Ping, Fuchu He, Changjun Wang, Gangqiao Zhou

**Affiliations:** 1 State Key Laboratory of Proteomics, National Center for Protein Sciences at Beijing, Beijing Institute of Radiation Medicine, Beijing, China; 2 Center for Disease Control and Prevention of PLA, Beijing, China; 3 Department of Laboratory Medicine, Wuhan Huoshenshan Hospital, Wuhan, Hubei China; 4 COVID-19 Research Center, Institute of Laboratory Medicine, Jinling Hospital, Nanjing University School of Medicine, Nanjing Clinical College of Southern Medical University, Nanjing, Jiangsu China; 5 State Key Laboratory of Proteomics, National Center for Protein Sciences at Beijing, Beijing Institute of Lifeomics, Beijing, China; 6 Union Hospital, Tongji Medical College, Huazhong University of Science and Technology, Wuhan, Hubei China; 7 School of Medicine, South China University of Technology, Guangzhou, Guangdong China; 8 Clinical Laboratory Medicine Center, Fifth Medical Center of Chinese PLA General Hospital, Beijing, China; 9 Medical College of Guizhou University, Guiyang, Guizhou China; 10 School of Public Health (Shenzhen), Sun Yat-sen University, Shenzhen, Guangdong China; 11 University of Chinese Academy of Sciences, Beijing, China; 12 Department of Otolaryngology, The First Medical Center of General Hospital of PLA, Beijing, China; 13 Guangzhou Laboratory, Guangzhou, Guangdong China; 14 Center for Global Health, School of Public Health, Nanjing Medical University, Nanjing, Jiangsu China

**Keywords:** Genome-wide association studies, Population genetics

## Abstract

Severe acute respiratory syndrome coronavirus 2 (SARS-CoV-2) infection causes a broad clinical spectrum of coronavirus disease 2019 (COVID-19). The development of COVID-19 may be the result of a complex interaction between the microbial, environmental, and host genetic components. To reveal genetic determinants of susceptibility to COVID-19 severity in the Chinese population, we performed a genome-wide association study on 885 severe or critical COVID-19 patients (cases) and 546 mild or moderate patients (controls) from two hospitals, Huoshenshan and Union hospitals at Wuhan city in China. We identified two loci on chromosome 11q23.3 and 11q14.2, which are significantly associated with the COVID-19 severity in the meta-analyses of the two cohorts (index rs1712779: odds ratio [OR] = 0.49; 95% confidence interval [CI], 0.38–0.63 for T allele; *P* = 1.38 × 10^−8^; and index rs10831496: OR = 1.66; 95% CI, 1.38–1.98 for A allele; *P* = 4.04 × 10^−8^, respectively). The results for rs1712779 were validated in other two small COVID-19 cohorts in the Asian populations (*P* = 0.029 and 0.031, respectively). Furthermore, we identified significant eQTL associations for *REXO2*, *C11orf71*, *NNMT*, and *CADM1* at 11q23.3, and *CTSC* at 11q14.2, respectively. In conclusion, our findings highlight two loci at 11q23.3 and 11q14.2 conferring susceptibility to the severity of COVID-19, which might provide novel insights into the pathogenesis and clinical treatment of this disease.

## Introduction

Recently, a novel coronavirus disease 2019 (COVID-19) caused by severe acute respiratory syndrome coronavirus 2 (SARS-CoV-2) is spreading rapidly throughout the world^1^, and has already affected over 110 million individuals worldwide, causing more than 2.5 million deaths (WHO, 2021, https://covid19.who.int; up to 8 March, 2021). Its rapid transmission, high infectivity, unpredictable clinical outcomes, and high mortality make COVID-19 a global threat^1^, but the pathogenesis of this disease is still not fully understood. The SARS-CoV-2 infection causes a broad spectrum of COVID-19 ranging from asymptomatic carrier, mild, moderate, severe, and critical pneumonia to death^2,3^. Like that of other major infectious diseases^4^ and another coronavirus disease — severe acute respiratory syndrome (SARS)^5,6^, the development of COVID-19 may also be the result of a complex interaction between the microbial, environmental, and host genetic components. Early reports have shown that elder age, male sex, and comorbid conditions (including hypertension, diabetes, and coronary artery diseases) are risk factors for the development of COVID-19^7–11^, but the extent to which genetic factors might influence susceptibility to the SARS-CoV-2 infection or disease severity remains largely unknown. Mapping the genetic variants that account for disease risk can identify disease pathways in complex disorders. Thus, a hope to resolve the etiology of COVID-19 has prompted a search for its candidate susceptibility genes.

To identify the genetic loci contributing to the COVID-19 severity and outcomes, a project of ‘COVID-19 Host Genetics Initiative’ was launched^12^. Up to 18 February 2021, five genome-wide genetic studies on COVID-19 have been published^13–17^. For examples, high-throughput sequencing studies have identified rare loss-of-function variants in *toll-like receptor 7* (*TLR7*) in Dutch severe COVID-19 patients^13^, and several genes involved in type I interferon (IFN) pathway in severe COVID-19 patients (most of whom are of European ancestry)^14^. Two genome-wide association studies (GWASs) in populations of European ancestry identified six loci to be significantly associated with COVID-19 severity^15,17^, including 3p21.31 (index single nucleotide polymorphism [SNP]: rs11385942), 9q34.2 (rs657152), 12q24.13 (rs10735079), 19p13.2 (rs74956615), 19p13.3 (rs2109069), and 21q22.1 (rs2236757). However, the allele and genotype frequencies of these SNPs vary among different populations; especially for rs11385942 and rs74956615 which are monomorphic in the Chinese population^15^, suggesting there may exist novel susceptibility loci to the development of COVID-19 in the Chinese population. Furthermore, the susceptibility for infectious diseases is considered to be determined at different functional levels^4^, suggesting an unknown number of other unidentified genes are likely to modify the susceptibility to COVID-19. Here, to identify novel loci for COVID-19 severity, we performed a GWAS of COVID-19 severity among the Chinese population.

## Results

### Genome-wide association analyses

**Fig. 1:**
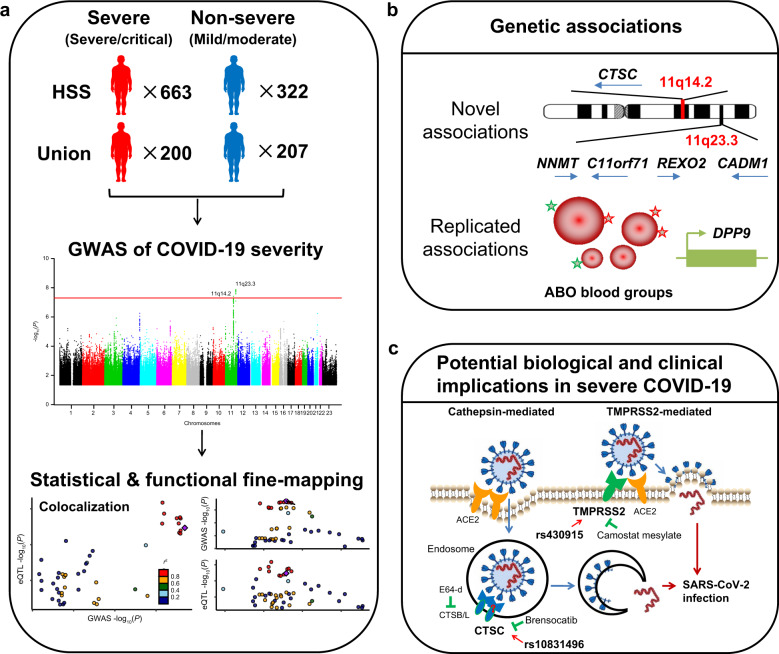
Summary of the GWAS for COVID-19 severity in the Chinese population. **a** Analyses pipeline of this study. We performed a genome-wide association study (GWAS) on 863 severe or critical COVID-19 patients (cases) and 529 mild or moderate patients (controls) from two hospitals, Huoshenshan (HSS) and Union hospitals, Wuhan city, Hubei province, China (after quality controls). We also conducted statistical and functional fine-mapping analyses for the two loci that reached the genome-wide significance (*P* = 5 × 10^−8^). eQTL, expression quantitative trait locus. **b** Main findings of this study. We identified two novel loci (chromosome 11q23.3 and 11q14.2) to be significantly associated with COVID-19 severity (*P* < 5 × 10^−8^). We also replicated two loci that were previously shown to be associated with COVID-19 (i.e. ABO blood groups at 9q34.2 and *dipeptidyl peptidase 9* [*DPP9*] at 19p13.3). **c** Potential biological and clinical implications in severe COVID-19 suggested by the genetic findings in this study. A recent study has shown that SARS-CoV-2 can use the endosomal cysteine proteases Cathepsin B and L (CTSB/L) and the serine protease transmembrane serine protease 2 (TMPRSS2) for cell entry (through endocytosis or membrane fusion mechanisms, respectively), and the combination use of the CTSB/L inhibitor (E64-d) and TMPRSS2 inhibitor (camostat mesylate) can fully block the entry of SARS-CoV-2 into human cells. In this study, we have suggested that *CTSC*, which encodes an endosomal cysteine protease, to be a candidate susceptibility gene of COVID-19 severity (index SNP rs10831496). A phase III clinical trial “STOP-COVID19” is running to evaluate whether the Cathepsin C (CTSC) inhibitor Brensocatib (DB15638) can help COVID-19 patients. In *TMPRSS2* gene, we also detected a suggestive association signal (index SNP rs430915).

To detect the genetic loci conferring susceptibility to COVID-19 severity in the Chinese population, we recruited 885 severe or critical COVID-19 patients (designated as cases) and 546 mild or moderate patients (designated as controls) from two cohorts (including Huoshenshan and Union cohorts) (Fig. 1). We then used the Affymetrix Axiom® World Arrays to genotype the 770,570 SNPs on these subjects. After stringent quality filtering (Methods section), a total of 558,642 SNPs were finally retained in 663 cases and 322 controls from the Huoshenshan cohort, and 200 cases and 207 controls from the Union cohort, respectively (Table 1, and Supplementary Table S1, Fig. S1).

**Table 1:** Clinical characteristics of the COVID-19 patients passed quality controls in this study.

Categories	Huoshenshan cohort		*P* values	Union cohort		*P* values
	Cases (*n* = 663)	Controls (*n* = 322)		Cases (*n* = 200)	Controls (*n* = 207)	
Age, years						
Mean (SD)	64.2 (13.7)	56.6 (13.9)	1.4 × 10^−15^	61.8 (14.0)	52.2 (16.2)	5.0 × 10^−10 ^
< 65, *n* (%)	305 (46.0)	214 (66.5)	2.5 × 10^−9^	106 (53.0)	160 (77.3)	4.5 × 10^−7^
≥ 65, *n* (%)	358 (54.0)	108 (33.5)	–	94 (47.0)	47 (22.7)	–
Gender, *n* (%)			0.88			0.082
Male	343 (51.7)	169 (52.5)	–	111 (55.5)	96 (46.4)	–
Female	320 (48.3)	153 (47.5)	–	89 (44.5)	111 (53.7)	–
Comorbidities, *n* (%)						
Hypertension	251 (37.9)	90 (28.0)	0.0027	77 (38.5)	62 (30.0)	0.042
Diabetes	119 (17.9)	37 (11.5)	0.012	37 (18.5)	26 (12.6)	0.13
Coronary artery diseases	61 (9.2)	14 (4.3)	0.010	28 (14.0)	9 (4.3)	0.0013
Chronic hepatitis B	16 (2.4)	9 (2.8)	0.89	3 (1.5)	1 (0.5)	0.36
Chronic obstructive pulmonary disease	14 (2.1)	1 (0.0)	0.028	6 (3.0)	0 (0.0)	0.014
Chronic renal diseases	6 (0.1)	2 (0.1)	1.00	3 (1.5)	0 (0.0)	0.12
Cancers	24 (3.6)	5 (1.6)	0.11	13 (6.5)	5 (2.4)	0.054
Symptoms, *n* (%)						
Fever	470 (70.9)	205 (63.7)	0.027	143 (71.5)	132 (63.8)	0.12
Cough	506 (76.3)	209 (64.9)	0.00022	142 (71.0)	123 (59.4)	0.014
Headache	15 (2.3)	3 (0.1)	0.20	13 (6.5)	6 (2.9)	0.14
Fatigue	343 (51.7)	152 (47.2)	0.21	96 (48.0)	68 (32.9)	0.0026
Pharyngalgia	15 (2.3)	7 (2.2)	1.00	7 (3.5)	7 (3.4)	1.00
Dyspnea	51 (7.7)	7 (2.2)	9.4 × 10^−4^	25 (12.5)	4 (1.9)	2.6 × 10^−5^
Death, *n* (%)	30 (4.5)	0 (0.0)	9.9 × 10^−6^	21 (10.5)	0 (0.0)	1.9 × 10^−7^

*SD* standard deviation.

Clinical classifications of COVID-19 patients were made according to the Chinese management guideline for COVID-19 (version 7.0). The severe and critical COVID-19 patients were defined as the “cases”, and the mild and moderate COVID-19 patients were defined as the “controls”. The *χ*^2^ test or fisher’s exact test was performed to compare the differences of clinical characteristics between the cases and controls, depending on the sample sizes. Fever was defined as axillary highest temperature of at least 37.3 °C.

**Fig. 2:**
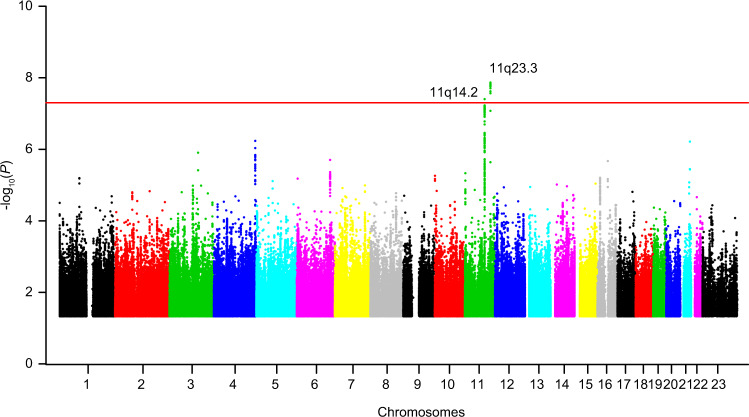
Manhattan plot of the meta-analyses association statistics highlighting two loci significantly associated with COVID-19 severity. The Manhattan plot showed the genome-wide association statistics from the main meta-analyses (adjusting for age, gender, comorbidities, and the top five principal components) of the Huoshenshan and Union cohorts. The red dashed line indicates the genome-wide significance threshold of *P* = 5 × 10^−8^. Two loci (11q14.2 and 11q23.3) reached this threshold, and were highlighted. The *x*-axis represents the genomic position (human genome assembly hg19), and the *y*-axis shows the −log_10_ (*P*).

After further imputation, we achieved 8,637,345 SNPs in Huoshenshan cohort and 8,557,677 SNPs in Union cohort, respectively (Supplementary Table S2). We then performed genome-wide association tests in these two cohorts, respectively, by the logistic regression model, with adjustment for age, gender, comorbidities (including hypertension, type 2 diabetes and coronary artery diseases), and the top five principal components (PCs) from principal component analysis (PCA) (designated as “main analyses”; Supplementary Fig. S2). Meta-analyses were then performed on the 8,227,125 SNPs that passed quality controls in both cohorts (Fig. 2). A quantile–quantile (Q–Q) plot suggested minimal overall inflation of the genome-wide statistical results (inflation factor *λ* = 1.038; Supplementary Fig. S2). We also performed genome-wide association tests adjusting for age, gender, comorbidities, and the top 10 or 20 principal components from PCA (designated as “analyses corrected for 10 or 20 PCs”, Supplementary Fig. S3).

### Two loci showed genome-wide significance

**Fig. 3:**
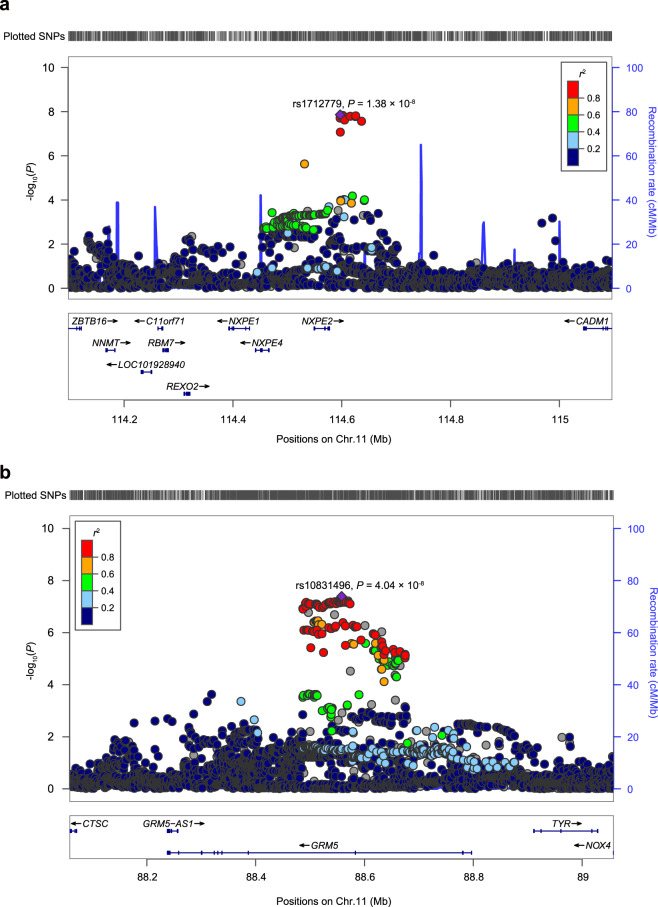
Regional plots for associations in the regions surrounding the rs1712779 and rs10831496 in the main meta-analyses. Results are shown for the SNPs within 1 mega bases (Mb) regions surrounding the index SNPs rs1712779 at Chr. 11q23.3 (**a**) and rs10831496 at Chr. 11q14.2 (**b**). The *P* values of the SNPs in these two regions in the main meta-analyses (adjusting for age, gender, and comorbidities) of Huoshenshan and Union cohorts are shown. Genomic positions are based on human genome assembly hg19. The *P* values of rs1712779 and rs10831496 are shown as purple diamonds. The linkage disequilibrium (LD) values (*r*^2^) to the index SNPs (rs1712779 or rs10831496) for the other SNPs are indicated by marker color. Red signifies *r*^2^ ≥ 0.8, with orange 0.6 ≤ *r*^2^ < 0.8, green 0.4 ≤ *r*^2^ < 0.6, light blue 0.2 ≤ *r*^2^ < 0.4, and blue *r*^2^ < 0.2. The estimated recombination rates in East Asian population (from the 1000 Genomes Project, November, 2014) are plotted in light blue. The East Asian population from the 1000 Genomes Project consists of 504 subjects from CHB (Han Chinese in Beijing, China), CHS (Southern Han Chinese), CDX (Chinese Dai in Xishuangbanna, China), JPT (Japanese in Tokyo, Japan), and KHV (Kinh in Ho Chi Minh City, Vietnam). Chr, chromosome; SNP, single-nucleotide polymorphism; cM, centi Morgan.

We identified two loci to be significantly associated with COVID-19 severity in the main meta-analyses, which reached the genome-wide significance threshold of *P* = 5 × 10^−8^: the chromosome 11q23.3 (index SNP rs1712779; odds ratio [OR] for the T allele = 0.49; 95% confidence interval [CI] = 0.38-0.63; *P* = 1.38 × 10^−8^), and the chromosome 11q14.2 (index SNP rs10831496; OR for the A allele = 1.66; 95% CI = 1.38-1.98; *P* = 4.04 × 10^−8^) (Table 2, Fig. 3, and Supplementary Fig. S4). These two index SNPs also showed nominally significant associations in both Huoshenshan and Union cohorts (all *P* < 0.001; Table 2). No evidence for heterogeneity of OR values for these two SNPs was observed across the two cohorts (*P*_heterogeneity_ = 0.53 and 0.63, respectively; Table 2). Comparing the frequencies of rs1712779 T and rs10831496 A alleles between the critical cases (*n* = 146) and controls (*n* = 529) in this study, we also found significant associations (*P* = 6.13 × 10^−4^ and OR = 0.51 for rs1712779, and *P* = 7.17 × 10^−4^ and OR = 1.77 for rs10831496, respectively). Intriguingly, the frequencies of rs1712779 T allele decreased with the increase of the disease severity (mild: 0.182, moderate: 0.164, severe: 0.092, critical: 0.084; *P* = 1.10 × 10^−9^, trend *χ*^2^ test; Supplementary Table S3). The rs10831496 was directly genotyped with high call rate (99%), and rs1712779 was imputed with high imputation quality (IMPUTE4 info score 0.98), suggesting the associations between these two loci and COVID-19 severity were unlikely to be false positives caused by the errors of genotyping or imputation. Furthermore, the analyses corrected for 10 or 20 PCs also achieved significant associations for these two index SNPs (*P* = 1.35 × 10^−8^ and 4.11 × 10^−9^ for rs1712779, and *P* = 6.89 × 10^−8^ and 4.42 × 10^−8^ for rs10831496, respectively; Supplementary Table S4).

**Table 2:** Association results for the rs1712779 and rs10831496 in the Huoshenshan and Union Cohorts in the main analyses.

SNPs	Chr. (cytoband)	Cohorts	Minor allele frequencies		ORs	*P* values	*P*_heterogeneity_
(Minor/major allele)			Cases^a^	Controls^b^	(95% CIs)		
rs1712779 (T/A)	11q23.3	Huoshenshan	0.100	0.166	0.52 (0.38–0.70)	1.58 × 10^−5^	0.53
		Union	0.082	0.166	0.44 (0.28–0.67)	1.85 × 10^−4^	
		Meta (in-house data)	0.096	0.166	0.49 (0.38–0.63)	1.38 × 10^−8^	
		Wang et al. study	0.081	0.113	0.31 (0.11–0.89)	0.029	0.092
		Erola et al. study (Asian)	NA	NA	0.78 (0.62–0.98)	0.031	
		Meta (external data)	NA	NA	0.75 (0.60–0.93)	0.010	
		Meta (all)	NA	NA	0.62 (0.52–0.73)	1.36 × 10^−8^	0.010
rs10831496 (A/G)	11q14.2	Huoshenshan	0.279	0.194	1.61 (1.29–2.00)	2.04 × 10^−5^	0.63
		Union	0.295	0.191	1.77 (1.28–2.43)	4.75 × 10^−4^	
		Meta (in-house data)	0.283	0.193	1.66 (1.38–1.98)	4.04 × 10^−8^	
		Wang et al. study	0.198	0.231	0.77 (0.42–1.42)	0.40	0.22
		Erola et al. study (Asian)	NA	NA	1.15 (0.93–1.43)	0.20	
		Meta (external data)	NA	NA	1.10 (0.90–1.35)	0.36	
		Meta (all)	NA	NA	1.38 (1.21–1.58)	2.31 × 10^−6^	0.0063

*Chr.* chromosome, *CI* confidence interval, *OR* odds ratio, *SNP* single-nucleotide polymorphism.

^a^The severe and critical types of COVID-19 patients were defined as the “cases”.

^b^The mild and moderate types of COVID-19 patients were defined as the “controls”.

After quality controls, the Huoshenshan cohort includes 663 cases and 322 controls, and the Union cohort includes 200 cases and 207 controls. In the main analyses, the associations in each cohort were calculated using logistic regression models adjusted for age, gender, comorbidities (including hypertension, type 2 diabetes, and coronary artery diseases) and the top five principal components. Meta-analyses of the association results generated from the Huoshenshan and Union cohorts were conducted using a fixed-effect model. ORs and 95% CIs are shown with respect to the minor alleles. The index rs1712779 at Chr. 11q23.3 was imputed with high confidence (IMPUTE4 info scores were 0.97 in the Huoshenshan cohort, and 0.98 in the Union cohort). The rs10831496 at Chr. 11q14.2 was genotyped by Affymetrix Axiom® World Arrays. The replication results presented here included the results of two other COVID-19 GWASs: (1) Wang et al. study (PMID: 33298875). This study contained 69 severe/critical COVID-19 patients (cases) and 215 mild/moderate COVID-19 patients (controls) from China, who were subjected to whole-genome sequencing. We only retained the subjects from Hubei Province, and achieved 43 cases and 127 controls. To increase power, 200 naïve controls from Hubei Province, as well as 662 genetically well-matched naïve controls according to CONVERGE (China, Oxford and Virginia Commonwealth University Experimental Research on Genetic Epidemiology) consortium (Changsha, *n* = 72; Chongqing, *n* = 295; Nanchang, *n* = 295) were also included. These naïve controls were subjected to whole-genome sequencing. Thus, 43 cases and 989 controls were used in the final association test. The association test was performed in logistic regression models adjusting for age and gender. (2) Erola et al. study (PMID: 33307546). This study included the populations of European ancestry (primary analyses), East Asian, South Asian, and African ancestries. Here we showed the results from the populations of the Asian ancestries (meta-analysis of East Asian ancestry and South Asian ancestry; 386 critically ill COVID-19 patients and 1930 naive controls).

Further, when adjusting for the effects of rs1712779 or rs10831496, no other SNPs on 11q23.3 or 11q14.2 locus showed evidence of association (all *P* > 0.001; Supplementary Fig. S5), suggesting that there might exist a single susceptibility locus on 11q23.3 or 11q14.2, respectively. Conditional analysis also showed that the associations at these two loci were independent of each other (Supplementary Methods). Additionally, no statistical interaction was observed between the index SNPs and the age, gender, and comorbidities (all *P* > 0.05; Supplementary Table S5), thus maximally ruling out the possibility that the detected association signals reflect the above-mentioned factors related to risk of COVID-19 severity.

We also assessed whether these two signals are significantly associated with other traits or diseases, using the PhenoScanner (www.phenoscanner.medschl.cam.ac.uk), which includes the GWAS catalog, the Genome-Wide Repository of Associations Between SNPs and Phenotypes (GRASP) database and other publicly available GWASs. We focused on the SNPs with *r*^2^ ≥ 0.8 to the index SNPs (rs1712779 or rs10831496) in East Asian or European population. Finally, we only found that rs10831496 was significantly associated with tanning/suntan in the European population (*P* < 5 × 10^−8^; Supplementary Table S6).

In addition to these two loci, a further 13 loci showed suggestive associations with COVID-19 severity in the main analyses (all *P* < 1 × 10^−5^; Supplementary Table S7).

### Chromosome 11q23.3 locus

The index SNP rs1712779 maps to an intergenic region on chromosome 11q23.3. The association signal at this locus was fine-mapped to 9 noncoding SNPs, which spans ~40 kilo bases (kb) (Supplementary Table S8). These 9 SNPs were annotated by use of RegulomeDB (V2), which includes chromatin immunoprecipitation (ChIP)-seq, formaldehyde-assisted isolation of regulatory elements (FAIRE), and DNase I hypersensitive site data from the Encyclopedia of DNA Elements (ENCODE) project. We found that rs11215172 has the highest functional probability score of 0.61 (Supplementary Table S8). Four of these 9 SNPs were predicted to be located at DNase I hypersensitive sites, including rs11215172 (Supplementary Table S8). rs11215172 was also predicted to be located at a binding site for ZNF146 (Supplementary Table S8). Together, these results indicated that several SNPs, especially the rs11215172, have potential functional relevance. However, more studies are needed to identify the causal SNP(s) at this locus.

The patients harboring the rs1712779 protective allele T (TT or TA; *n* = 303) showed significantly lower C-reactive protein (medium: 3.15 mg/L) and higher lymphocyte counts (medium: 1.36 × 10^−9^/L), compared with those of other patients (AA; *n* = 1,057) (medium: 5.22 mg/L and 1.25 × 10^−9^/L, respectively; *P* = 0.0044 and 0.050, respectively).

Nine protein-coding genes are located within 1 mega bases (Mb) region surrounding the rs1712779, including *ZBTB16*, *NNMT*, *C11orf71*, *RBM7*, *REXO2*, *NXPE1*, *NXPE4*, *NXPE2*, and *CADM1* (Fig. 3a). To identify the potentially causative gene(s) at 11q23.3, we performed expression quantitative trait locus (eQTL) association analyses using several publicly available data sets (Methods section). The protective allele T of rs1712779 showed significant associations with higher expression of *REXO2* and *C11orf71* in whole blood tissues according to the QTLbase (all *P* < 0.05; Supplementary Table S9)^18^, even after Bonferroni correction for multiple comparisons (*n* = 9). These eQTL signals were further replicated in adrenal gland or heart tissues from the Genotype-Tissue Expression (GTEx) database, and in monocytes from the ImmunPop (all *P* < 0.05; Supplementary Table S9)^19^. Several other genes in this region, such as *NNMT* and *CADM1*, also showed significant eQTL associations after accounting for the multiple testing in adipose or adrenal gland from the GTEx database (*P* = 0.00052 and 0.0046, respectively; Supplementary Table S9). We further performed colocalization analyses for GWAS and eQTL signals. Although no gene passed the threshold of 80% posterior probability which was deemed to be “highly likely to colocalize” in these data sets, *NNMT* (posterior probability = 65%) was identified as the top gene in the colocalization analyses (Supplementary Methods; data not shown). These results suggested that *NNMT* is most likely to be the potential candidate gene at this locus. However, other three genes with the eQTL evidence (*CADM1*, *REXO2*, and *C11orf71*) may also be the potential candidate genes, and the causative gene cannot be reliably implicated by the present data.

### Chromosome 11q14.2 locus

The rs10831496 is located at an intronic region of *GRM5* gene on 11q14.2. The association signal at this locus was fine-mapped to 67 noncoding SNPs (Supplementary Table S8), spanning ~115 kb. Using RegulomeDB (V2), we found that four SNPs (rs11021570, rs7109255, rs586927, and rs1813119) have high probability scores of >0.8 (Supplementary Table S8). These four candidate SNPs were all located at transcription factor binding sites, and three ones (rs7109255, rs586927, and rs1813119) were at DNase I hypersensitive sites (Supplementary Table S8). Data from the Roadmap Epigenomics Project also showed that three (rs11021570, rs7109255, and rs1813119) out of the four SNPs were at the predicted enhancers in multiple cells/tissues, such as primary T cells and liver tissues (Supplementary Table S8). Together, these pieces of evidence suggested that several SNPs at this locus were likely to be the causal variants.

The patients harboring the rs10831496 risk allele A (AA or GA; *n* = 591) showed significantly lower platelet counts (medium: 188 × 10^−9^/L), compared with that of other patients (GG; *n* = 789) (medium: 196 × 10^−9^/L; *P* = 0.0040).

Four protein-coding genes are located within 1 Mb region surrounding the rs10831496, including *CTSC*, *GRM5*, *TYR*, and *NOX4* (Fig. 3b). The risk allele A of rs10831496 is significantly associated with higher expression of *Cathepsin C* (*CTSC*) gene in the whole blood tissues, CD16^+^ neutrophils and naive CD4^+ ^T cells according to the QTLbase (the most significant *P* = 5.4 × 10^−30^; Supplementary Table S9)^18^. This eQTL signal was further replicated in esophagus and muscle tissues from the GTEx database (*P* = 0.0014 and 0.0095, respectively), in whole blood tissues from Lepik’s study^20^ (*P* = 0.0090; Supplementary Table S9) and peripheral blood mononuclear cells (PBMCs) from Pierce’s study^21^ (*P* = 0.00034; Supplementary Table S9). Intriguingly, the colocalization analyses showed that the COVID-19-associated SNP rs10831496 is colocalized with eQTL signals of *CTSC* in a population of South Asian ancestry^21^ (Supplementary Fig. S6 and Supplementary Methods). No significant eQTL association was found between rs10831496 and the other three genes at this region (all *P* > 0.05). Taken together, these results suggested a potential role for *CTSC* in the development of COVID-19.

### Association tests of previously reported SNPs and genes

Recently, two GWASs in European populations has identified six loci that were genome-wide significantly associated with severe COVID-19, including 3p21.31 (index rs11385942), 9q34.2 (rs657152), 12q24.13 (rs10735079), 19p13.2 (rs74956615), 19p13.3 (rs2109069), and 21q22.1 (rs2236757)^15,17^. The SNPs rs11385942 and rs74956615 are found to be monomorphic in the Chinese population, and therefore not included in this study. The SNP rs2109069 within *dipeptidyl peptidase 9* (*DPP9*) gene showed a significant association with COVID-19 severity in the Huoshenshan cohort (*P* = 0.032, OR = 1.33 for A allele; Supplementary Table S10). When adding the naïve controls (*n* = 954; Methods section), the rs2109069 in *DPP9* gene and rs657152 in *ABO* gene were significantly associated with COVID-19 severity (for rs2109069 A allele: *P* = 0.0042, OR = 1.33; for rs657152 A allele: *P* = 0.0017, OR = 1.27).

Additionally, a genetic association study of COVID-19 severity in the Chinese population reported two suggestive signals^16^, including *TMEM189-UBE2V* (index rs6020298) and *TMPRSS2* (index rs12329760) loci. In the present study, the rs6020298 in *TMEM189-UBE2V* was not associated with COVID-19 severity (*P* = 0.72, OR = 0.97 for A allele), while the rs12329760 in *TMPRSS2* showed a nominal association (*P* = 0.010, OR = 0.81 for T allele) (Supplementary Table S11).

We also sought to test the associations between ABO blood groups and the severity of COVID-19. We predicted ABO blood groups from combinations of genotypes of two SNPs (rs529565 and rs8176746)^22^. In the meta-analyses, we found that the blood groups A and O are not associated with the severity of COVID-19 when correcting for multiple tests (adjusted *P* > 0.05; Supplementary Table S12). When adding the naïve controls (*n* = 954) in the association test, we found that the blood group A was significantly associated with higher risk of the severity of COVID-19 (OR = 1.22, *P* = 3.2 × 10^−4^; Supplementary Table S12), and the blood group O was significantly associated with lower risk of severity of COVID-19 (OR = 0.83, *P* = 1.5 × 10^−3^; Supplementary Table S12). These results were consistent with those identified in severe COVID-19 patients with respiratory failure in European populations^15^.

Our and others’ previous studies have ever reported several SNPs that were associated with the SARS infection or severity^5,23,24^. Here, we observed that rs2071430 and rs17000900 in the promoter of *myxovirus resistance 1* (*MX1*), a key effector of interferon^23^, also show significant associations with the COVID-19 severity (*P* = 3.8 × 10^−6^ and *P* = 0.0050, respectively, in the main analysis; Supplementary Table S13). These results suggested that there might exist shared genetic risk factors among diseases caused by different types of coronavirus infections.

*Human leukocyte antigen* (*HLA*) genes were well-known as important candidate susceptibility genes for multiple infectious diseases^25^. Thus, we interrogated the SNPs in Major Histocompatibility Complex (MHC) regions (human genome assembly hg19, Chr.6:28,477,898 - 33,448,354). However, no SNPs in this region showed significant association with the COVID-19 severity in this study (all *P* > 0.001). We also explored the associations between the *HLA* classical alleles and the COVID-19 severity. We performed the *HLA* allele genotyping in silico on the basis of the known SNPs genotypes (Supplementary Methods). No *HLA* alleles showed significant association signal in both Huoshenshan and Union cohorts (Supplementary Table S14). These results were consistent with those of the recent GWAS of severe COVID-19 with respiratory failure in Italian and Spanish populations^15^. However, several *HLA* alleles showed nominal associations with the COVID-19 severity in Huoshenshan or Union cohort (*P* < 0.05; Supplementary Table S14), including B*58:01 and C*3:02 alleles, which also showed nominal associations with COVID-19 severity in the Italy population, but showed no associations in the Spanish population^15^. Further studies are needed to verify these observations.

## Discussion

In the present study, we conducted a GWAS on genetic susceptibility to COVID-19 severity in the Chinese population, and identified two novel loci at chromosome 11q23.3 and 11q14.2. Here, we selected the controls from those who were mild/moderate COVID-19 patients, but not from the general population. This may avoid the type II error (false-negative error)^5^. Because the uninfected subjects from general population remain at risk developing to severe/critical COVID-19, the inclusion of such a control group limits the ability to obtain positive results when comparing contributions of SNPs in control subjects with those in patients with severe/critical COVID-19. Because this is a hospital-based study, the COVID-19 patients who showed mild symptoms and did not need hospitalization were not included here. This may raise the concern that the pathological differences between the cases and the controls may be relatively subtle. However, when analyzing the immunological and biochemical parameters, we found substantial differences between the cases and controls (Supplementary Table S15). Additionally, our GWAS reveals an enough statistical power in 863 cases and 529 controls to detect the index rs1712779 and rs10831496, with estimated powers to be ~95% and ~81%, respectively (Supplementary Fig. S7).

We further extracted the genotypes for these two indices SNPs from another Chinese genetic study of COVID-19 severity (Methods section)^16^. This initial study also used the severe/critical COVID-19 patients as cases, and the symptomatic/mild/moderate patients and naïve controls as controls. We only retained the genetically matched subjects, and achieved 43 cases and 989 controls (Methods section). We found that the rs1712779 T allele showed nominally significant association with COVID-19 severity (*P* = 0.029, OR = 0.31; Table 2 and Supplementary Table S16). Further, we used another Asian COVID-19 cohort, which consisted of 386 critically ill COVID-19 patients and 1930 naïve controls of East Asian and South Asian ancestries from a recent COVID-19 study^17^. We used rs1712791 A allele instead of rs1712779 T allele (*r*^2^ = 1 and *D*’ = 1 with rs1712779 T allele in the Chinese population; *r*^2^ = 0.99 and *D*’ = 1 with rs1712779 T allele in the East Asian population; *r*^2^ = 0.92 and *D*’ = 0.96 with rs1712779 T allele in the South Asian population), as rs1712779 was not included. Again, we found that the rs1712779 T allele also showed nominally significant association with COVID-19 severity in Asian populations (*P* = 0.031, OR = 0.78; Supplementary Table S16). The rs1712779 T allele showed a more significant association in meta-analysis of external data (*P* = 0.010, OR = 0.75; Table 2), and remained genome-wide significant in meta-analysis using both in-house and external data (*P* = 1.36 × 10^−8^, OR = 0.62; Table 2), which added the evidence to support our findings. However, no significant association was found for rs10831496 in the Asian populations of these two studies (all *P* > 0.05; Table 2 and Supplementary Table S16). These results may be due to the limited sample size of Asian cases in these two studies (*n* = 43 and 386, respectively). Interestingly, another SNP rs12276517 G allele (*r*^2^ = 0.59 and *D*’ = 0.98 with rs10831496 A allele in Asian populations) showed nominally significant association with high risk of COVID-19 severity in Asian populations^17^ (*P* = 0.046, OR = 1.24). We further performed meta-analysis in the Asian populations (both in-house and external data) on the two reported loci based on the LD blocks. At 11q23.3 locus, the index SNP rs1712779 remained the lead SNP, which showed a genome-wide significant association with the risk of COVID-19 severity (*P* = 1.36 × 10^−8^, OR = 0.62 for T allele). At 11q14.2 locus, we found that the lead SNP is rs6483475 (*P* = 1.63 × 10^−6^, OR = 1.39 for A allele). This SNP showed strong LD with the index SNP rs10831496 (*P* = 2.31 × 10^−6^, OR = 1.38 for A allele) in the Asian populations (*r*^*2*^ = 0.98). We did not find any novel signal in meta-analyses of the Asian populations. The associations between these two index SNPs and COVID-19 severity were not significant in European GWASs (all *P* > 0.05; Supplementary Table S16), which may be due to the different study designs or racial diversities. For example, the controls in this study were mild/moderate COVID-19 patients, while the controls in the previous European GWASs were naïve controls^15,17^; and the cases in this study were severe/critical COVID-19 patients, while the cases in the European GWASs were more severe cases (i.e. severe COVID-19 with respiratory failure or severe COVID-19 in intensive care units)^15,17^. Besides, the non-significant associations in the European populations may also be due to the differences in genetic architecture across ethnicities. Indeed, at the LD block tagged by rs10831496 (Supplementary Methods), the most significant signal in the European populations of Erola et al. study^17^ was rs592381 (*P* = 7.2 × 10^−4^, OR = 1.16 for A allele). The A allele of rs592381 was also significantly associated with COVID-19 severity in other European populations of Ellinghaus et al. study^15^ and COVID-19 hg (A2_ALL) (*P* = 0.032 and 0.0038, OR = 1.12 and 1.09, respectively). The rs592381 A allele showed low LD with the index rs10831496 A allele (*r*^2^ = 0.14, *D*’ =0.75 in the European populations, and *r*^2^ = 0.005, *D*’ =0.47 in the Asian populations). However, the rs592381 A allele (risk allele in European populations) also showed significant association with higher expression of *CTSC* in whole blood tissues (*P* = 1.2 × 10^−7^) from QTLbase, which also suggested a risk role for *CTSC* in the COVID-19 severity. Another potential complication causing the inconsistent association results across ethnic groups may be the gene–gene interactions as well as gene-environment interactions that vary among populations^26^.

We observed that the frequency of index rs1712779 T allele at 11q23.3 in COVID-19 patients was similar to that in naïve Chinese controls (*n* = 954), which have no information on SARS-CoV-2 infection status (Supplementary Table S17). When compared with the genotypes of rs1712779 in five main populations from the 1000 Genomes Project (*n* = 2504), the rs1712779 T allele frequency in our COVID-19 patients was similar to that of population from the East Asian ancestry, and significantly less than that of South Asian, African, American, and European ancestry (all *P* < 0.05, *χ*^2^ test; Supplementary Table S17). The frequency of rs10831496 A allele in our COVID-19 patients was similar to that of naïve controls and East Asian descent, slightly higher than that of African descent, and significantly less than that of South Asian, Americans and Europeans (all *P* < 0.05, *χ*^2^ test; Supplementary Table S17). Further studies are needed to check whether the differences of these two SNPs’ frequencies among different ethnic populations influence susceptibility to the COVID-19 severity.

These two loci are biologically plausible for susceptibility to the COVID-19 severity. On 11q23.3 locus, the eQTL analyses suggested four candidate genes (*REXO2*, *C11orf71*, *NNMT*, and *CADM1*). Among these four genes, *cell adhesion molecule 1* (*CADM1*) has been shown to be potentially relevant to COVID-19. This gene was mainly expressed in IgG/IgA peripheral blood B cells (Supplementary Fig. S8a)^27,28^, and has been reported to promote immune surveillance^29^. However, the eQTL analyses are complicated due to tissue heterogeneity; cautions should be applied when interpreting the eQTL data. We further checked the single-cell RNA-sequencing data of nasopharynx tissues^27^, and found that the expressions of RNA binding motif protein 7 (*RBM7*) in cells from nasopharynx tissues (such as neutrophils) are significantly higher in critical COVID-19 patients than those in moderate patients (adjusted *P* < 0.05; Supplementary Fig. S9). RBM7 has been shown to trigger apoptosis of lung epithelial cells^30^. The other genes at 11q14.2 locus may also be relevant to COVID-19, such as *zinc finger and BTB domain containing 16* (*ZBTB16*). ZBTB16 has been shown to play critical roles in the development and function of natural killer T cells and innate lymphoid cells^31–33^.

At 11q14.2, the possible candidate gene is *CTSC*. This gene encodes an endosomal cysteine protease (also known as dipeptidyl peptidase 1, DPP1), which plays important roles in activating many serine proteinases in immune cells^34^. *CTSC* was mainly expressed in blood, spleen, and lung tissues according to the GTEx database. Especially, *CTSC* was expressed in dendritic cells and CD16^+^ monocytes in PBMCs (Supplementary Fig. S8b)^27,28^. The expressions of *CTSC* in cells from nasopharynx tissues (such as non-resident macrophages) were significantly higher in critical COVID-19 patients than in moderate patients (adjusted *P* < 0.05; Supplementary Fig. S9)^27^. The roles of CTSC in modulating the inflammatory responses have been widely investigated^35–37^. For examples, in *Ctsc*-knocked-out mouse models of chronic obstructive pulmonary disease (COPD) and sepsis, the inflammatory cell infiltrations and proinflammatory cytokine productions were significantly decreased^35,36^. Clinically, CTSC has been considered to be a potential target in inflammation therapy^38^. More intriguingly, a phase III clinical trial “STOP-COVID19” is running to evaluate whether the CTSC (DPP1) inhibitor Brensocatib (DB15638) could help COVID-19 patients (https://stop-covid19.org.uk/). Taken together, these pieces of evidence suggested a strongly biological plausibility for CTSC in the development of COVID-19. However, several other gene(s) at 11q14.2 locus may also be relevant to COVID-19, such as *NADPH Oxidase 4* (*NOX4*), which has been reported to be involved in inflammation by activating oxidative stress^39^. Further studies are needed to explore the causal gene(s) at these two loci.

A recent study has reported that SARS-CoV-2 can use the endosomal cysteine proteases (Cathepsin B and L, CTSB/L) and the serine protease transmembrane serine protease 2 (TMPRSS2) for cell entry (through endocytosis and membrane fusion, respectively), and the combination uses of the CTSB/L inhibitor (E64-d) and TMPRSS2 inhibitor (camostat mesylate) can fully block the entry of SARS-CoV-2 into human cells^40^. Intriguingly, in *TMPRSS2* gene, we also detected a suggestive association signal (index rs430915, OR for the A allele = 1.38; *P* = 1.4 × 10^−4^; Supplementary Table S18). The eQTL analyses showed that rs430915 risk allele A is significantly associated with higher expressions of *TMPRSS2* in lung tissues from the GTEx (v8) and another lung eQTL study^41^ (*P* = 6.7 × 10^−8^ and *P* = 2.8 × 10^−13^, respectively). Although the associations between rs430915 and COVID-19 severity were not significant in European GWASs (all *P* > 0.05; Supplementary Table S18), we found another SNP rs11911394, which was in moderate LD with rs430915 (*r*^2^ = 0.30 and 0.38 in the East Asian population and European population, respectively), showed significant associations in this study and other GWASs in the Asian and European populations (all ORs for the C allele <1; all *P* < 0.05; Supplementary Table S18). These results suggested that the genetic variants in *TMPRSS2* may also be responsible for the risk of the COVID-19 severity.

Across two cohorts, the index rs1712779 at 11q23.3 and rs10831496 at 11q14.2 were associated with a population attributable fraction (PAF) of 17.4% and 19.1%, respectively (Methods section), suggesting a relatively low fraction of the risk of developing severe/critical COVID-19 in the Chinese population studied here. Thus, an unknown number of other genes are likely to modify the susceptibility to COVID-19 severity.

In conclusion, our GWAS provides evidence for two previously unidentified susceptibility loci contributing to the COVID-19 severity on 11q23.3 and 11q14.2 (Fig. 1). Several candidates involved in inflammation and immune cell functions were identified, such as *CTSC*, *CADM1*, *REXO2*, and *ZBTB16*. Our findings implicate that the different immune response to the virus determined by host genetic diversity may be an important factor in determining the severity of COVID-19. Finally, potential therapeutic targets were implicated in the current study, including *CTSC*, which may benefit the treatment of COVID-19. Further functional studies are warranted to establish the roles of these two loci in the pathogenesis of COVID-19.

## Materials and methods

### Study participants and recruitment

This study included 1431 adult hospitalized COVID-19 patients recruited from two hospitals at Wuhan city, Hubei province, China: (1) Huoshenshan hospital (*n* = 1010; designated as the Huoshenshan cohort) between 4 February 2020 and 23 March 2020; and (2) Union hospital of Tongji Medical College of Huazhong University of Science & Technology (*n* = 421; designated as the Union cohort) between 15 January 2020 and 30 March 2020. The diagnosis of COVID-19 was made by a confirmed SARS-CoV-2 infection from nasopharyngeal swabs using real-time reverse transcriptase polymerase chain reaction (RT-PCR) assay in these two hospitals. Clinical classifications of COVID-19 patients (mild, moderate, severe, or critical) were made according to the Diagnosis and Treatment Protocol for COVID-19 (version 7.0). Briefly, the COVID-19 patients were divided into four categories: mild, moderate, severe, and critical types. The mild COVID-19 patients were those patients with mild clinical symptoms, and had no sign of pneumonia on chest imaging. The moderate COVID-19 patients were those patients having fever, respiratory symptoms, and radiological evidence of pneumonia. The severe COVID-19 patients were those patients having at least one of the following three conditions: respiratory distress, respiratory rate ≥ 30 beats/minute; means oxygen saturation ≤ 93% in a resting state; and arterial blood oxygen partial pressure/oxygen concentration ≤ 300 mm Hg. The critical COVID-19 patients were those patients having at least one of the following three conditions: shock incidence; respiratory failure and requiring mechanical ventilation; and admission to intensive care unit (ICU) with other organ function failure. The classifications of COVID-19 severity were taken as the worst classification during the patient’s hospital stay. These assessments were done by at least two physicians. Three of the authors (Y.L., Y.W., and X.L.) further verified the assessments. In this study, to identify the susceptibility loci contributing to severity of COVID-19, the severe and critical COVID-19 patients were defined as the “cases”, and the mild and moderate COVID-19 patients were defined as the “controls”. Thus, we achieved a total of 679 cases and 331 controls in the Huoshenshan cohort, and 206 cases and 215 controls in the Union cohort, respectively. After quality controls, 663 cases (566 severe and 97 critical patients) and 322 controls (322 moderate patients) in the Huoshenshan cohort, and 200 cases (151 severe and 49 critical patients) and 207 controls (11 mild and 196 moderate patients) in the Union cohort were finally retained for subsequent association tests. Because this is a hospital-based study, the COVID-19 patients who showed mild symptoms and did not need hospitalization were not included here. Thus, the percentage of severe/critical patients in this study (~60%) is much higher than that among all infections (~15%)^42^. All of the 11 mild and 518 moderate patients were cured and discharged from the hospital, 4 severe patients died of COVID-19 (0.56%, 4/717), and 47 critical patients died of COVID-19 (32.19%, 47/146). The clinical characteristics of the patients in these two cohorts were extracted from the electronic medical records. Data were reviewed and verified by experienced respiratory physicians from each hospital. Three of the authors (Y.L., Y.W., and X.L.) further crossly checked the data. We collected three broad classes of characteristics (Table 1): (1) demographic variables (age, sex, and ethnicity); (2) symptoms (fever, cough, headache, fatigue, pharyngalgia, and dyspnea); and (3) comorbid conditions (hypertension, diabetes, coronary artery diseases, chronic hepatitis B, chronic obstructive pulmonary disease, chronic renal diseases, and cancer). Comorbidities listed here are defined as medical diagnoses included in medical history by International Classification of Diseases (ICD)-10 coding.

For later validation of allele frequencies of the significantly associated SNPs in the naïve controls of Chinese ancestry, we additionally collected a total of 954 samples. For detailed descriptions, see Supplementary Methods.

This study was performed with the approval of the Medical Ethical Committee of Beijing Institute of Radiation Medicine (Beijing, China) (reference number, AF/SC-08/02.57) and the Medical Ethical Committee of Union Hospital in Tongji Medical College in Huazhong University of Science & Technology (Wuhan city, China) (reference number, 2020/0075-01). Written informed consent was obtained, sometimes in a delayed fashion, from the study patients when possible. In some instances, informed consent was provided verbally or by the next of kin, depending on local ethics committee regulations.

### Genotyping, quality controls, and principal component analysis

Peripheral whole blood samples were collected from all participants (679 cases and 331 controls in the Huoshenshan cohort, and 206 cases and 215 controls in the Union cohort). Genomic DNAs were extracted from 1 mL of peripheral whole blood, according to the manufacturer’s instructions (QIAamp DNA blood kits, Qiagen, Crawley, UK). The purified DNA was eluted in 100 µL of elution buffer (pH 8.0). The quality of the isolated genomic DNA was verified by the following two methods: (1) the DNA degradation and contamination were monitored on 1% agarose gels; and (2) the DNA concentration was measured using a Qubit DNA Assay Kit and a Qubit 2.0 Fluorometer (Life Technologies, MA, USA).

Genotyping for these subjects was performed using the Affymetrix Axiom® World Arrays, which consist of 770,570 SNPs, at CapitalBio Corporation (Beijing, China). Genotype callings were performed using Axiom Analysis Suite (Version 3.1.51) based on the default workflow. Affymetrix adopts a “dish quality control” (DQC) score of >0.82 for internal quality control, and all samples in this study passed this quality control. Overall, 97.45% and 97.53% of the genotypes were called in the Huoshenshan cohort and Union cohort, respectively.

We then performed stringent quality controls for both samples and SNPs to ensure subsequent robust association tests. Because the same genotyping platform was used, we were able to perform uniform quality controls for the merged SNP data of these two cohorts. As for the samples quality controls, among the Huoshenshan cohort, five individuals (3 cases and 2 controls) were excluded as they had call rates < 90%; 5 individuals (4 cases and 1 control) were excluded as they showed sex discrepancies; and 14 individuals (9 cases and 5 controls) were excluded as they were related with the other individuals based on pairwise identity-by-state by “PI_HAT” values in PLINK (PI_HAT > 0.5 and between 0.25 and 0.5 indicates the first and second degree relatives, respectively). One control was identified as an outlier using principal component analysis (the first two principal components were outside of 5 standard deviations of the population mean). Among the Union cohort, one case with call rate < 90%, 1 case with sex discrepancy, and 12 individuals (4 cases and 8 controls) who were related with other individuals were excluded. As for the SNPs quality controls, the SNPs that were not in chromosomes 1–23 or X (*n* = 8,734), had call rates <90% among all subjects in this study (*n* = 12,155), deviated significantly from Hardy–Weinberg equilibrium (HWE) among all subjects in this study (*P* < 0.0001; *n* = 14,003) or had minor allele frequencies (MAF) < 1% among all subjects in this study (*n* = 177,036) were excluded from subsequent analyses. Thus, a total of 558,642 SNPs in 663 cases and 322 controls in the Huoshenshan cohort, and 200 cases and 207 controls in the Union cohort were finally retained, with an average call rate of 98.6% and 98.9%, respectively (Supplementary Table S1).

To identify the ancestry outliers, we performed principal component analysis (PCA) on the two cohorts by EIGENSOFT (v3)^43^. We used the autosomal SNPs for PCA based on the following criteria: call rate >90%, Hardy–Weinberg *P* > 0.0001, MAF > 10% and linkage disequilibrium (LD)-pruned *r*^2^ < 0.10. Twenty principal components were estimated for all the cases and controls. Moreover, we also performed PCA based on the same SNPs for all samples from the 1000 Genomes Project (Phase 3, November 2014; *n* = 2,504). Using PCA, we observed that all the cases and controls passed quality controls were genetically well matched, and were of Chinese ancestry. We also performed PCA of the COVID-19 patients and naïve controls in this study and the reference populations from the 1000 Genomes Project. We confirmed that all the naïve controls are of Chinese ancestry (data not shown).

### Imputation analyses

To increase the coverage of genomic region, we performed imputation on the GWAS genotyping data using SHAPEIT (v2)^44^ and IMPUTE (v4)^45^. First, a prephasing strategy for the Huoshenshan and Union cohorts was performed by SHAPEIT, using the 1000 Genomes Project data (Phase 3, November, 2014) as the reference based on the human genome assembly hg19. Then, IMPUTE4 was used to impute the phased haplotypes constructed by SHAPEIT. For the imputation of chromosome X, we coded males as diploid in non-pseudoautosomal regions. The imputation was performed separately for the Huoshenshan cohort and Union cohort. The cases and controls within each cohort were imputed together. After imputation, the SNPs with IMPUTE4 info scores below 0.6 or MAF < 0.01 were excluded. Finally, we achieved a total of 8,637,345 SNPs in the Huoshenshan cohort and 8,557,677 SNPs in the Union cohort, respectively (Supplementary Table S2). We also performed an independent imputation analyses using the Westlake Imputation Server (https://imputationserver.westlake.edu.cn/), a Chinese population specific imputation server^46^. The imputation was performed separately for the Huoshenshan cohort and Union cohort. The cases and controls within each cohort were imputed together. After imputation, the SNPs with R-squares (Rsqs) below 0.6 or MAF < 0.01 were excluded. Finally, a total of 7,513,512 SNPs passed quality controls in both cohorts. After genome-wide association tests and meta-analyses, we did not find any novel signal reached genome-wide significant association.

### Genome-wide association tests

To account for imputation uncertainty, case–control allele-dose association tests of the genotyped and imputed SNPs in the two cohorts were performed separately, using SNPTEST software (logistic regression)^47^. We carried out genome-wide association tests in logistic regression models adjusting for covariates including: age (“age ≥ 65” coded as 1, and “age < 65” coded as 0), gender (“male” coded as 1, and “female” coded as 0), comorbidities (including hypertension, type 2 diabetes, and coronary artery diseases, “diagnosed” coded as 1, and “not diagnosed” coded as 0) and the top five principal components from PCA (designated as “main analyses”). We also performed genome-wide association tests adjusting for age, gender, comorbidities, and the top 10 or 20 principal components from PCA (designated as “analyses corrected for 10 or 20 PCs”). The quantile–quantile plot was constructed using the R-package script. A lambda (*λ*) inflation factor is given to indicate whether the systematic bias is present. Adjusting for 5 PCs may be sufficient to account for the population substructures in this study, based on the following reasons: (1) GWASs typically use 5–20 PCs to correct for population structures^48^. (2) The COVID-19 patients in this study were all recruited from one city (Wuhan city). Thus, the confounding effects of population structure in this study may be very weak. (3) The quantile–quantile plot and inflation factor *λ* (1.038) suggested minimal overall inflation of the genome-wide statistical results in the analyses corrected for 5 PCs. The λ inflation factors even increased slightly in the analyses corrected for 10 or 20 PCs, compared with that in the analyses corrected for 5 PCs.

### Expression quantitative trait locus analyses

To identify the potentially causative gene(s) at the significantly associated loci, we performed eQTL analyses using four publicly available datasets, which provided a user-friendly access to the results: (1) QTLbase^18^, which contains 233 independent QTL studies across 78 types of human tissues/cells (including whole blood tissues, CD16^+^ neutrophils, naive CD4^+^ T cells, and lung tissues). (2) Genotype-Tissue Expression (GTEx, version 8), which contains 48 types of tissues (including blood and lung tissues). The SNPs were detected using whole-genome sequencing. The mRNA expression levels were quantified using RNA sequencing. (3) ImmunPop QTL browser^19^, which contains monocytes from 134 healthy males of German ancestry. The SNPs were genotyped using the Illumina’s HumanOmniExpress BeadChips. The mRNA expression levels were detected using the Human HT-12 v4 Expression BeadChips, over a detailed time course of 90 min (min) and 6 h (h) following stimulation with lipopolysaccharide (LPS), muramyl-dipeptide (MDP), or in vitro transcribed 5′-ppp-dsRNA. Thus, seven conditions (baseline, LPS90min, LPS6h, MDP90min, MDP6h, RNA90min, and RNA6h) were available for eQTL analyses. (4) Lepik’s study^20^, which contains whole blood tissues from 491 Estonian subjects. The SNPs were detected using whole-genome sequencing. The mRNA expression levels were quantified using RNA sequencing. (5) Pierce’s study^21^, which contains PBMCs from 1799 Bangladeshi subjects. The SNPs were detected using the Illumina HumanCytoSNP-12 v2.1 BeadChip. The mRNA expression levels were quantified using the Illumina HumanHT-12 v4.0 Gene Expression BeadChip. We only focused on the 9 and 4 protein-coding genes within 1 Mb region surrounding the rs1712779 and rs10831496, respectively. The *P* value after Bonferroni correction for multiple comparisons (*n* = 9 and 4 for rs1712779 and rs10831496, respectively) of < 0.05 was considered to be statistically significant.

### Bayesian fine-mapping analyses and SNPs annotation

A Bayesian fine-mapping analysis was carried out using three tools, including the PAINTOR (v3.0), CAVIARBF (v.0.2.1), and FINEMAP (v.1.3), to determine a credible set of SNPs for each locus, i.e., a minimum set of variants containing all causal variants with certainty >0.95%. To this end, we calculated the local LD structure in a collection of East Asian population from the 1000 Genomes Project reference panel (defined by the 1000 Genomes Project, consisting of 504 subjects from CHB [Han Chinese in Beijing, China], CHS [Southern Han Chinese], CDX [Chinese Dai in Xishuangbanna, China], JPT [Japanese in Tokyo, Japan], and KHV [Kinh in Ho Chi Minh City, Vietnam]), which served together with the meta-analyses summary statistics as an input for the fine-mapping analysis. We set the option -- n-causal-snps as 1. The causal SNPs were defined as those predicted to be candidate causal SNPs by all these three tools. Finally, the association signals at 11q23.3 and 11q14.2 were fine-mapped to 9 and 67 SNPs, respectively (Supplementary Table S8).

We also used RegulomeDB (V2) to identify the potentially functional SNPs. RegulomeDB is a database that annotates SNPs with known and predicted regulatory elements. RegulomeDB (V2) includes chromatin immunoprecipitation (ChIP)-seq, formaldehyde-assisted isolation of regulatory elements (FAIRE), and DNase I hypersensitive site data sets from the Encyclopedia of DNA Elements (ENCODE) project. We used the 9 SNPs at 11q23.3 and the 67 SNPs at 11q14.2 as inputs, and the query SNPs were included in the outputs of RegulomeDB.

### Association analyses for rs1712779 and rs10831496 in other COVID-19 GWASs

To replicate our GWAS results, we inquired the results of four other COVID-19 GWASs from three published articles and an online public resource: (1) Wang et al. study^16^. This study contained 69 severe/critical COVID-19 patients (cases) and 215 mild/moderate COVID-19 patients (controls) from China, who were subjected to whole-genome sequencing. We only retained the subjects from Hubei Province, and achieved 43 cases and 127 controls. To increase power, 200 naïve controls from Hubei Province, as well as 662 genetically well-matched naïve controls from CONVERGE (China, Oxford and Virginia Commonwealth University Experimental Research on Genetic Epidemiology) consortium (Changsha, *n* = 72; Chongqing, *n* = 295; Nanchang, *n* = 295) were also included. These naïve controls were also subjected to whole-genome sequencing. Thus, 43 cases and 989 controls were used in the final association test. The association test was performed in logistic regression models adjusting for age and gender. (2) Ellinghaus et al. study^15^. This study included 1610 severe COVID-19 patients with respiratory failure and 2205 naïve controls from Italy and Spain, who were genotyped by Global Screening Array (GSA) (v2). The association test was performed in logistic regression models adjusting for the top 10 principal components. (3) Erola et al. study^17^. This study included the populations of European ancestry (primary analyses), East Asian, South Asian, and African ancestries. Here we showed the results from the populations of European (1676 critically ill COVID-19 patients and 8379 naive controls) and the Asian ancestries (meta-analysis of East Asian ancestry and South Asian ancestry; 386 critically ill COVID-19 patients and 1930 naive controls). (4) COVID-19 hg (release 4) (an online public resource accessed from https://www.covid19hg.org/results/). We checked the results of “very severe respiratory confirmed covid vs. not hospitalized covid” (A1_ALL) (269 cases and 688 controls in European populations) and “very severe respiratory confirmed covid vs. population controls without known SARS-CoV-2 infection” (A2_ALL) (4933 cases and 1,398,672 controls in European and American populations). We also performed meta-analyses of the GWAS summary results of the Asian populations and European populations separately on the two loci (11q23.3 and 11q14.2). The GWAS summary results of the Asian populations were obtained from two COVID-19 GWASs: Wang et al. study^16^ and Erola et al. study^17^. The GWAS summary results of the European populations were obtained from three COVID-19 GWASs: Ellinghaus et al. study^15^, Erola et al. study^17^, and COVID-19 hg.

### The population attributable fraction and power calculation

The population attributable fraction (PAF) was estimated by the formula^49^: PAF % =100% × (*x* – 1)/*x*, where *x* = (1−*p*)^2^ + 2*p*(1−*p*)OR_1_ + p^2^OR_2_, *p* is the population allele frequency, and OR_1_ and OR_2_ are the ORs associated with heterozygosity and homozygosity, respectively. In this study, *p* is 0.166 for the protective T allele of rs1712779, and OR_1_ and OR_2_ are 0.543 and 0.224, respectively. Thus, PAF (rs1712779) = 17.4%. For the risk A allele of rs10831496, *p* is 0.193 and OR_1_ and OR_2_ are 1.483 and 3.312, respectively. Thus, PAF (rs10831496) = 19.1%.

Power analysis was performed using the Power for Genetic Association Analyses (PGA), which is a package of algorithms and graphical user interfaces developed in Matlab for power and sample size calculation under various genetic models and statistical constraints^50^. Giving the severe/critical COVID-19 prevalence of 15%, 863 cases and 529 controls, and *P* value of 5.0 × 10^−8^, the power of our GWAS to identify the rs1712779 at 11q23.3 (OR = 0.49; MAF = 0.166) is ~95%, and the power to rs10831496 at 11q14.2 (OR = 1.66; MAF = 0.193) is ~81%.

### Statistical analyses

The *χ*^2^ test, Fisher’s exact test or Mann–Whitney *U* test was performed to compare the differences of clinical characteristics between the cases and controls. We calculated the Cochran Q statistic to test for between-group heterogeneity, and the heterogeneity was considered to be statistically significant when *P* < 0.05. A fixed-effect model was used in the meta-analyses using Meta software (v1.7). We used the genome-wide significance threshold of *P* = 5 × 10^−8^ in meta-analyses to determine the statistical significance. To check whether there exist a single or multiple association signals in one locus, we performed logistic regression analyses conditional on the most strongly associated SNP at that locus.

### Other analyses

Details of the external control datasets, conditional analysis, LD block analysis, colocalization analyses of eQTLs and GWAS associations, ABO blood groups, *human leukocyte antigen* (*HLA*) alleles association tests, and single-cell RNA-sequencing analyses are provided in the Supplementary Methods.

## Supplementary information

Supplementary information

Tables S1-18

## Data Availability

The accession number for the genome-wide association data in this paper is CNP0002125 (China National GeneBank (CNGB)). All code and materials used in the analysis will be available from the corresponding author by request, for purposes of reproducing or extending the analysis.
