# Supplementary material for: Genome-wide association study of COVID-19 severity among the Chinese population
Source: Cell Discov. 2021 Aug 31;7:76. doi: 10.1038/s41421-021-00318-6 (PMC8408196; doi:10.1038/s41421-021-00318-6)
Supplement: Supplementary file 1 — Supplementary information [file 41421_2021_318_MOESM1_ESM.pdf]

Supplementary information

Genome-wide association study of COVID-19 severity among the Chinese population

Yuanfeng Li[1,†], Yuehua Ke[2,13,†], Xinyi Xia[3,13,†], Yahui Wang[1,5,†], Fanjun Cheng[6,†], Xinyi Liu[1,†], Xin Jin[7,†], Boan Li[8], Chengyong Xie[9], Siyang Liu[10], Weijun Chen[11], Chenning Yang[1], Yuguang Niu[12], Ruizhong Jia[2], Yong Chen[2], Xiong Liu[2], Zhihua Wang[2], Fang Zheng[6], Yan Jin[6], Zhen Li[6], Ning Yang[8], Pengbo Cao[1], Hongxia Chen[1], Jie Ping[1], Fuchu He[5,13], Changjun Wang[2,14,*] & Gangqiao Zhou[1,4,9,13,*]

[1]State Key Laboratory of Proteomics, National Center for Protein Sciences at Beijing, Beijing Institute of Radiation Medicine, Beijing, China;

[2]Center for Disease Control and Prevention of PLA, Beijing, China;

[3]COVID-19 Research Center, Institute of Laboratory Medicine, Jinling Hospital, Nanjing University School of Medicine, Nanjing Clinical College of Southern Medical University, Nanjing City, Jiangsu Province, China;

[4]Center for Global Health, School of Public Health, Nanjing Medical University, Nanjing City, Jiangsu Province, China;

[5]State Key Laboratory of Proteomics, National Center for Protein Sciences at Beijing, Beijing Institute of Lifeomics, Beijing, China;

[6]Union Hospital, Tongji Medical College, Huazhong University of Science and

Technology, Wuhan City, Hubei Province, China;

[7]School of Medicine, South China University of Technology, Guangzhou City, Guangdong Province, China;

[8]Clinical Laboratory Medicine Center, Fifth Medical Center of Chinese PLA General Hospital, Beijing, China;

[9]Medical College of Guizhou University, Guiyang City, Guizhou Province, China;

[10]School of Public Health (Shenzhen), Sun Yat-sen University, Shenzhen City, Guangdong Province, China

[11]University of Chinese Academy of Sciences, Beijing, China;

[12]Department of Otolaryngology, the First Medical Center of General Hospital of PLA, Beijing, China;

[13]Guangzhou Laboratory, Guangzhou City, Guangdong Province, China;

[14]Department of Laboratory Medicine, Wuhan Huoshenshan Hospital, Wuhan City, Hubei Province, China.

[†]These authors contributed equally.

[*]**Corresponding Authors:**

Dr. Gangqiao Zhou, State Key Laboratory of Proteomics, National Center for Protein Sciences at Beijing, Beijing Institute of Radiation Medicine, 27 Taiping Road, Beijing, 100850, P. R. China. E-mail: zhougq114@126.com; Phone & fax: 86-10-66931201.

OR

Dr. Changjun Wang, Center for Disease Control and Prevention of PLA, Beijing,

100071, P. R. China. E-mail: science2008@hotmail.com; Phone & fax: 86-10-66948307.

Index

Supplementary Methods

Supplementary Figures:

Supplementary Fig. S1 The principal components analyses of the COVID-19 patients in this study and the reference populations from the 1,000 Genomes Project.

Supplementary Fig. S2 Manhattan plots and quantile-quantile plots for the genome-wide associations of the Huoshenshan and Union cohorts in the main analyses.

Supplementary Fig. S3 Manhattan plots and quantile-quantile plots for the genome-wide associations in analyses corrected for 10 or 20 PCs.

Supplementary Fig. S4 Regional plots for the associations in the main analyses in regions surrounding the rs1712779 and rs10831496, respectively, in the Huoshenshan and Union cohorts, respectively.

Supplementary Fig. S5 Regional plots for the associations in regions surrounding the rs1712779 or rs10831496 when adjusting for the effect of rs1712779 or rs10831496.

Supplementary Fig. S6 Colocalization analyses of association signals from the eQTLs and GWAS at rs10831496 locus.

Supplementary Fig. S7 Power to detect the genetic effects of various sizes *versus* sample sizes.

Supplementary Fig. S8 Expression levels of the candidate genes at 11q23.3 and 11q14.2 in peripheral blood mononuclear cells and lung tissues.

Supplementary Fig. S9 Different expression patterns for the candidate genes at 11q23.3 and 11q14.2 in nasopharynx tissues from the critical and moderate COVID-19 patients.

Supplementary Tables:

Supplementary Table S1 Summary of the quality controls for samples and SNPs.

Supplementary Table S2 Summary of the SNP imputation.

Supplementary Table S3 The frequencies of rs1712779 and rs10831496 in mild, moderate, severe and critical COVID-19 patients.

Supplementary Table S4 Association results for rs1712779 and rs10831496 in Huoshenshan and Union Cohorts in the analyses corrected for 10 or 20 PCs.

Supplementary Table S5 Stratification analyses of rs1712779 and rs10831496 by age, gender and comorbidities.

Supplementary Table S6 Association results for the SNPs at 11q23.3 and 11q14.2 loci in previous GWASs.

Supplementary Table S7 Association results for SNPs showing suggestive associations.

Supplementary Table S8 The fine-mapping results for the chromosome 11q23.3 and 11q14.2 loci.

Supplementary Table S9 The expression quantitative trait locus (eQTL) results for rs1712779 and rs10831496.

Supplementary Table S10 Association results for the previously reported COVID-19-associated SNPs in the European population.

Supplementary Table S11 Association results for the previously reported COVID-19-associated SNPs in the Chinese population.

Supplementary Table S12 Association results for the ABO blood groups.

Supplementary Table S13 Association results for the previously reported SARS-associated SNPs.

Supplementary Table S14 Association results for the *HLA* alleles.

Supplementary Table S15 Immunological and biochemical parameters in severe/critical and mild/moderate COVID-19 patients.

Supplementary Table S16 Association results for rs1712779 and rs10831496 in other COVID-19 GWASs.

Supplementary Table S17 The allele and genotype frequencies of rs1712779 and rs10831496 in naïve controls of Chinese ancestry and in populations from the 1,000 Genomes Project.

Supplementary Table S18 Association results for the SNPs in *TMPRSS2* in this study and other COVID-19 GWASs.

Supplementary Methods

External control datasets

For later validation of allele frequencies of the significantly associated SNPs in the naïve controls of Chinese ancestry, we additionally collected two datasets, which contains a total of 954 subjects (designated as external control datasets 1 and 2, n = 298 and 656, respectively). The SARS-CoV-2 infection statuses of these subjects were unknown. External control dataset 1 contains 298 healthy male individuals who were recruited between March, 2018 and September, 2019 from Bengbu city at Anhui province in Northern China. The mean age (s.d.) of these individuals was 23.4 (1.6). These subjects have been genotyped previously using the Illumina Infinium Asian Screening Array-24 (v1.0) (Unpublished, in-house data). External control dataset 2 was derived from a previous study (*i.e.*, the controls in "GWAS population 1" in that study)[1]. This control dataset contains 656 healthy males recruited in 2009 from Fangchenggang city at Guangxi province in Southern China. The mean age (s.d.) of these individuals was 37.1 (10.8). These subjects were genotyped by Illumina Omini one array[1].

Conditional analysis

To check whether the associations at 11q23.3 (index SNP rs1712779) and 11q14.2 (index SNP rs10831496) loci are independent each other, we performed conditional logistic regression analysis using SNPTEST software. When adjusting rs10831496, as well as age, gender, comorbidities and top five principal components, the index SNP rs1712779 also showed significant associations in both Huoshenshan and Union

cohorts ($P = 4.33 \times 10^{-5}$ and 1.58×10^{-4}, respectively). When adjusting rs1712779, as well as age, gender, comorbidities and top five principal components the index SNP rs10831496 also showed significant associations in both Huoshenshan and Union cohorts ($P = 3.71 \times 10^{-5}$ and 6.23×10^{-5}, respectively). These results suggested that the associations at these two loci were independent each other.

LD block analysis

The LD blocks results were downloaded from https://bitbucket.org/nygcresearch/ldetect-data, which were computed by Pickrell et al.[2]. The LD block dataset is available for three populations: African, East Asian and European populations. In East Asian population, rs1712779 was in a 1.30 mega bases (Mb) LD block (Chr.11:114,123,539-115,428,159), and rs10831496 was in a 2.67 Mb LD block (Chr.11:88,282,078-90,954,609). In European population, rs1712779 was in a 0.57 Mb LD block (Chr.11:114,257,728-114,830,666). and rs10831496 was in a 1.78 Mb LD block (Chr.11:87,430,235-89,208,936).

Identification of genomic loci showing suggestive associations

Genomic loci showing suggestive associations with the severity of COVID-19 ($P < 1 \times 10^{-5}$) were identified using the functional mapping and annotation of genetic associations (FUMA)[3]. We used the core function of FUMA (the SNP2GENE process) to identify the "independently significant SNPs". First, the "independently significant SNPs" were defined as those ones with $P < 1 \times 10^{-5}$ and are independent from each other (LD $r^2 < 0.6$) by FUMA. These "independently significant SNPs" were further represented by "lead SNPs", which were a subset of the independent significantly

associated SNPs that are in approximate LD with each other at $r^2 < 0.1$. Based on the "independently significant SNPs", the associated 'genomic loci' were also defined by tagging all variants that were in LD ($r^2 \geq 0.6$) with at least one of the "independently significant SNPs". If the 'genomic loci' of independent significant variants were close (< 250 kilo bases [kb] based on the closest boundary variants), they were merged to a single genomic locus. We then defined the associated genomic risk loci by merging any physically overlapping lead SNPs. Finally, each locus is represented by the top lead SNP that has the minimum *P* value at that locus. In total, we found 13 top lead SNPs in 13 loci (Supplementary Table S7). The r^2 was calculated based on East Asian populations from the 1,000 Genomes Project reference panel (defined by the 1,000 Genomes Project, consisting of 504 subjects from CHB [Han Chinese in Beijing, China], CHS [Southern Han Chinese], CDX [Chinese Dai in Xishuangbanna, China], JPT [Japanese in Tokyo, Japan] and KHV [Kinh in Ho Chi Minh City, Vietnam]).

Colocalization analyses of eQTLs and GWAS associations

To identify the colocalization events between the eQTL and GWAS signals, we performed the colocalization analyses using the coloc.abf function which was implemented in the R package "coloc" (v3.2.1). The eQTL summary statistics were pre-computed by others (for example, eQTLs in South Asian population were computed by Pierce's study[4]). The GWAS summary statistics were from this study. These two types of the summary statistics were used as the input for "coloc". The colocalization analysis was run on all SNPs within 250 kb of the index SNPs (rs1712779 or rs10831496). Evidence for colocalization was assessed using the

posterior probability (PP) for five exclusive hypotheses: H0, neither trait has a genetic association in the region; H1/H2, only one trait has a genetic association in the region; H3, both traits are associated but with different causal variants; and H4, both traits are associated and share a single causal variant. Associations with a posterior probability of hypothesis 4 (PP4) > 0.8 was deemed to be "highly likely to colocalize", while PP4 > 0.5 was deemed to be "likely to colocalize with moderate evidence". The LocusCompare R package further helps to visualize the colocalization events, which generates a combined plot with two locus-zoom plots (eQTL and GWAS in the same gene region) and a locus-compare scatter plot (an eQTL $-\log_{10}(P)$ plot and a GWAS $-\log_{10}(P)$ plot). The figure indicates whether the GWAS top SNP is also the leading SNP in the eQTL result. In this study, we found that only the COVID-19-associated SNP rs10831496 was colocalized with the eQTL signals of *CTSC* in a South Asian population (PP4 score = 0.91).

ABO blood group analyses

To test associations between the ABO blood groups and severity of COVID-19, we first predicted ABO blood groups from combinations of genotypes of two SNPs (rs529565 and rs8176746)[5]. The genotypes of these two SNPs and the corresponding ABO blood groups are listed below (the individuals with OO were assigned blood group O; individuals with BO and BB were assigned blood group B; individuals with AO and AA were assigned blood group A; and individuals with AB were assigned blood group AB, respectively):

	rs529565		
	T/T	T/C	C/C

	T/T	N.D.	BO	BB
rs8176746	T/G	BO	BO	AB
	G/G	OO	AO	AA

N.D., no data.

To assess the accuracy of ABO blood prediction, we randomly selected 292 subjects from the Huoshenshan cohort, and determined the ABO blood groups by enzyme-linked immunosorbent assay (ELISA). Excellent concordance was observed between the array typing and the prediction (287/292 = 98.3%).

We further calculated the A, B, AB and O blood group frequencies in cases (n = 863), controls (n = 529) and naïve controls (n = 954) in this study. A logistic regression was conducted, adjusting for age, gender, comorbidities (hypertension, type 2 diabetes and coronary artery diseases) and the top five principal components when not using the naïve controls, and adjusting for age, gender and the top five principal components when using the naïve controls (as the information of the comorbidities is not available for naïve controls). The *P* value of less than 0.05 was considered to be statistically significant.

***HLA* alleles analyses**

To test the associations between the *HLA* alleles and severity of COVID-19, we performed *HLA* alleles imputation and association test in Huoshenshan and Union cohorts. For *HLA* alleles imputation, *HLA* alleles were predicted from dense SNPs genotypes using the R package HIBAG (http://cran.r-project.org/web/packages/HIBAG/index.html). The 1,000 Genomes Project data (Phase 3; containing 162 African, 193 American, 260 East Asian and 322 European samples) and IKMB dataset (containing 312 African, 162 German, 140

Chinese, 143 Indian, 132 Iranian, 189 Japanese, 122 South Korean and 160 Maltese samples) were used as the reference based on human genome assembly hg19. We selected a threshold of 0.5 as a value that has modest effects on both call rate and accuracy. For *HLA* alleles association test, odds ratios (ORs) and 95% confidence intervals (CIs) were calculated in logistic regression model under the additive model, adjusting for age, gender, comorbidities (hypertension, type 2 diabetes and coronary artery diseases) and the top five principal components. The P value of less than 0.05 was considered to be statistically significant.

We observed that no *HLA* allele shows a significant association with COVID-19 severity in both Huoshenshan and Union cohorts (Supplementary Table S14) or met even the significance threshold of suggestive association $P < 1\times10^{-5}$ in our meta-analysis. In fact, this result was consistent with the results from the previous COVID-19 studies[6,7]. In those studies, no *HLA* allele showed a suggestive association with COVID-19 severity.

Currently, there is no consistency regarding the possible associations between the *HLA* alleles and susceptibility to the SARS-CoV-2 infection and/or COVID-19 severity[8,9]. The lack of consistency among the recalled reports could be reasonably explained. Indeed, several methodological problems can elucidate these discrepancies: (i) insufficient sample sizes to detect the differences in *HLA* antigen frequencies; (ii) inadequate inclusion with inappropriate mixing of data (cohort effect) and inappropriate control matching, *i.e.*, lack of proper selection from the same population; (iii) the different genetic backgrounds of the populations; and (iv) the lack of

Bonferroni’s correction for multiple comparisons. Moreover, caution should be adopted in the attempt to justify the observed correlation between the *HLA* allele geographical distribution and COVID-19 incidence, prevalence and related mortality[8,9]. Collectively, these findings suggested that further large-scale studies will be required to determine the associations between the *HLA* alleles and COVID-19 severity.

Single cell RNA-sequencing datasets analyses

To check the gene expression distributions across cells in peripheral blood mononuclear cells (PBMCs) and lung tissues for the candidate genes at 11q23.3 and 11q14.2, we used two 10x Genomics single cell RNA-sequencing (scRNA-seq) datasets[10,11]. The PBMCs dataset consists of 28,094 cells from 8 PBMC samples from 7 COVID-19 patients, and the lung tissues dataset consists of 38,658 cells from 2 COVID-19 patients. To reveal the different expression patterns for the candidate genes at 11q23.3 and 11q14.2 between the critical COVID-19 patients and moderate COVID-19 patients/healthy individuals, we used a 10x Genomics scRNA-seq dataset from nasopharynx tissues of upper airway[10]. This dataset consists of 135,600 cells from 24 donors (including 11 critical COVID-19 patients, 8 moderate COVID-19 patients and 5 healthy individuals). The differential expression analyses were performed using the FIndmarkers function in Seurat (v3.0) between the critical and moderate COVID-19 patients. P value adjustment was performed using Bonferroni correction based on the total number of analyzed genes, and adjusted $P < 0.05$ and fold change [FC] $\geqslant$ 1.5 was considered to be statistically significant.

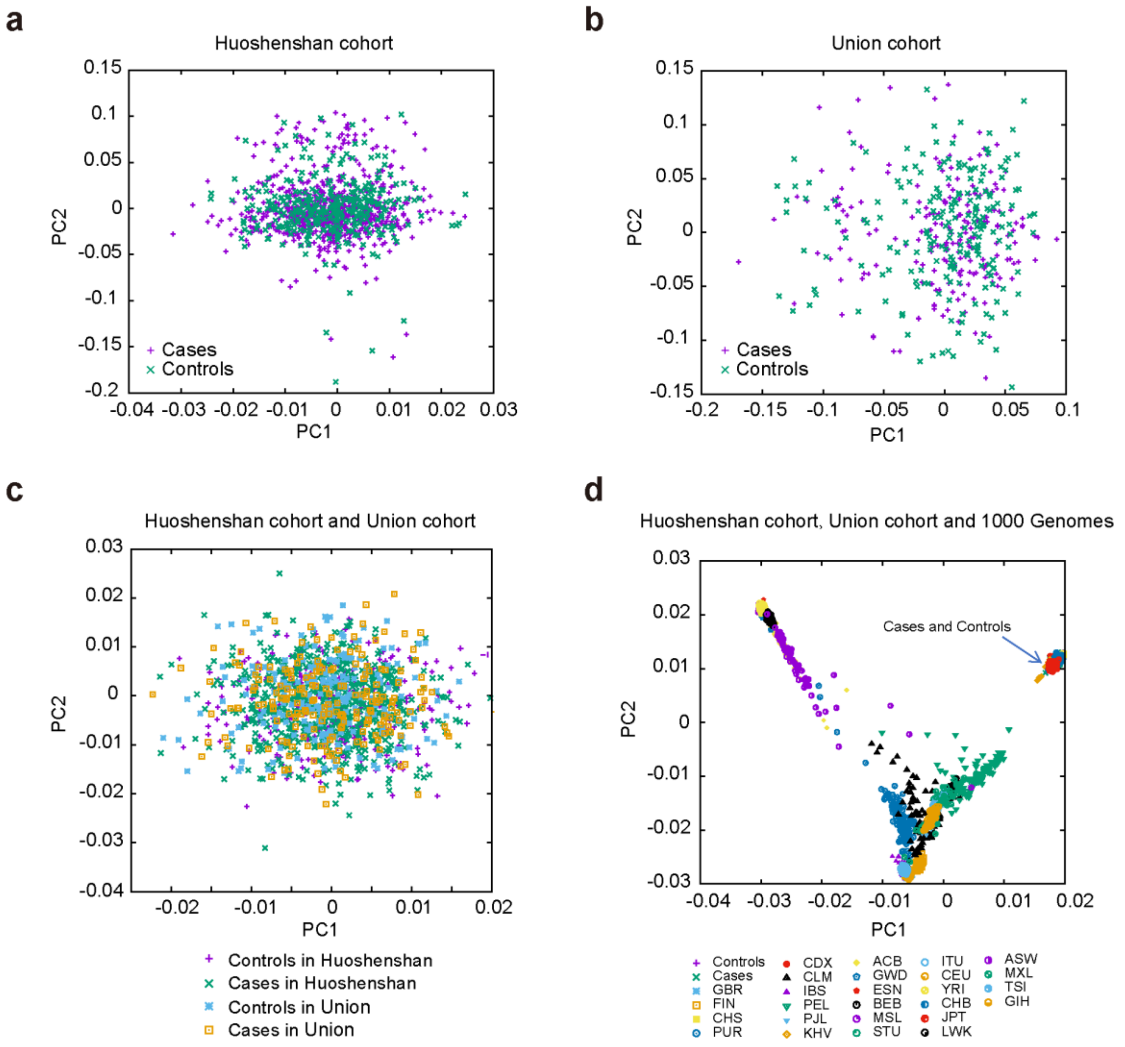

Supplementary Fig. S1 The principal components analyses of the COVID-19 patients in this study and the reference populations from the 1,000 Genomes Project. a Principal component (PC) 1 *versus* PC2 for the cases (n = 663) and controls (n = 322) in Huoshenshan cohort. **b** PC1 *versus* PC2 for the cases (n = 200) and controls (n = 207) in Union cohort. **c** PC1 *versus* PC2 for the cases (n = 863) and controls (n = 529) in the combined Huoshenshan and Union cohorts. **d** PC1 *versus* PC2 for the COVID-19 patients in this study (863 cases and 529 controls), and for the reference individuals in the 1,000 Genomes Project (Phase 3, November, 2014) (n = 2,504; consisting of 26 individual populations across the world). Purple represents the COVID-19 patients in this study. CHB, Han Chinese in Beijing, China (n = 103);

CHS, Southern Han Chinese (n = 105); CDX, Chinese Dai in Xishuangbanna, China (n = 93); JPT, Japanese in Tokyo, Japan (n = 104); KHV, Kinh in Ho Chi Minh City, Vietnam (n = 99); CEU, Utah Residents (CEPH) with Northern and Western European Ancestry (n = 99); TSI, Toscani in Italia (n = 107); FIN, Finnish in Finland (n = 99); GBR, British in England and Scotland (n = 91); IBS, Iberian Population in Spain (n = 107); YRI, Yoruba in Ibadan, Nigeria (n = 108); LWK, Luhya in Webuye, Kenya (n = 99); GWD, Gambian in Western Divisions in the Gambia (n = 113); MSL, Mende in Sierra Leone (n = 85); ESN, Esan in Nigeria (n = 99); ASW, Americans of African Ancestry in SW USA (n = 61); ACB, African Caribbeans in Barbados (n = 96); MXL, Mexican Ancestry from Los Angeles, USA (n = 64); PUR, Puerto Ricans from Puerto Rico (n = 104); CLM, Colombians from Medellin, Colombia (n = 94); PEL, Peruvians from Lima, Peru (n = 85); GIH, Gujarati Indian from Houston, Texas (n = 103); PJL, Punjabi from Lahore, Pakistan (n = 96); BEB, Bengali from Bangladesh (n = 86); STU, Sri Lankan Tamil from the UK (n = 102); ITU, Indian Telugu from the UK (n = 102).

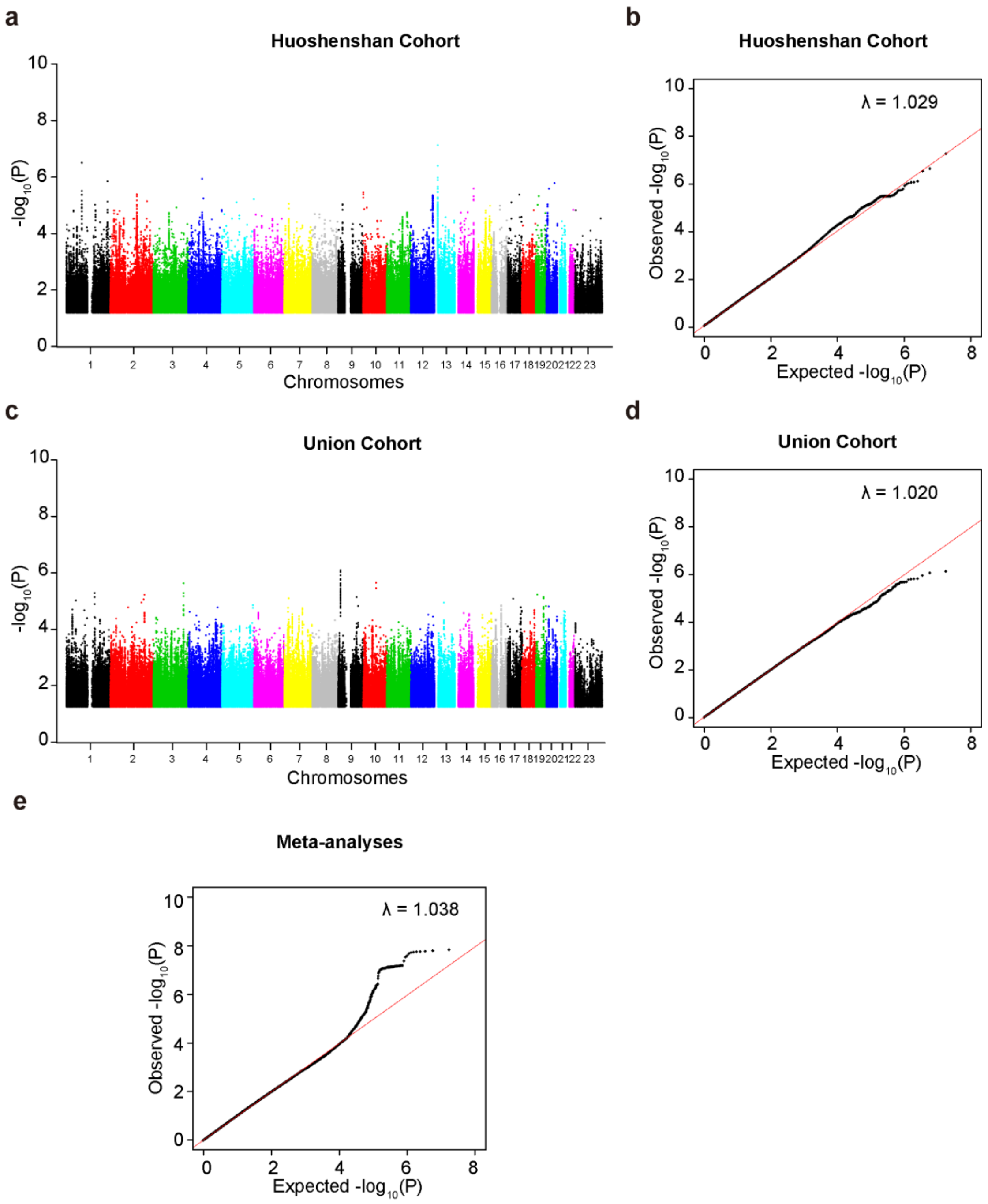

Supplementary Fig. S2 Manhattan plots and quantile-quantile plots for the genome-wide associations of the Huoshenshan and Union cohorts in the main analyses. a,c The Manhattan plots showing the genome-wide association statistics for the genotyped and imputed SNPs in the Huoshenshan (**a**) and Union cohorts (**c**), using logistic regression analyses under the additive model, adjusting for age, gender, comorbidities (including hypertension, type 2 diabetes and coronary artery diseases)

and the top five principal components (main analyses). The x-axis represents the genomic position (based on human genome assembly hg19), and the y-axis shows the $-\log_{10}(P)$. **b,d,e** The quantile-quantile plots for the Huoshenshan cohort (**b**), Union cohort (**d**) and the meta-analyses combining these two cohorts (**e**). Meta-analyses of the associations generated from the Huoshenshan and Union cohorts were conducted using a fixed-effect model. The red line represents the null hypothesis of no true association. The genomic inflation factor lambda (λ) is defined as the ratio of the medians of the sample χ^2 test statistics and the 1-d.f. χ^2 distribution (0.455). The λ values were 1.029 in the Huoshenshan cohort, 1.020 in the Union cohort, and 1.038 in the meta-analyses, respectively.

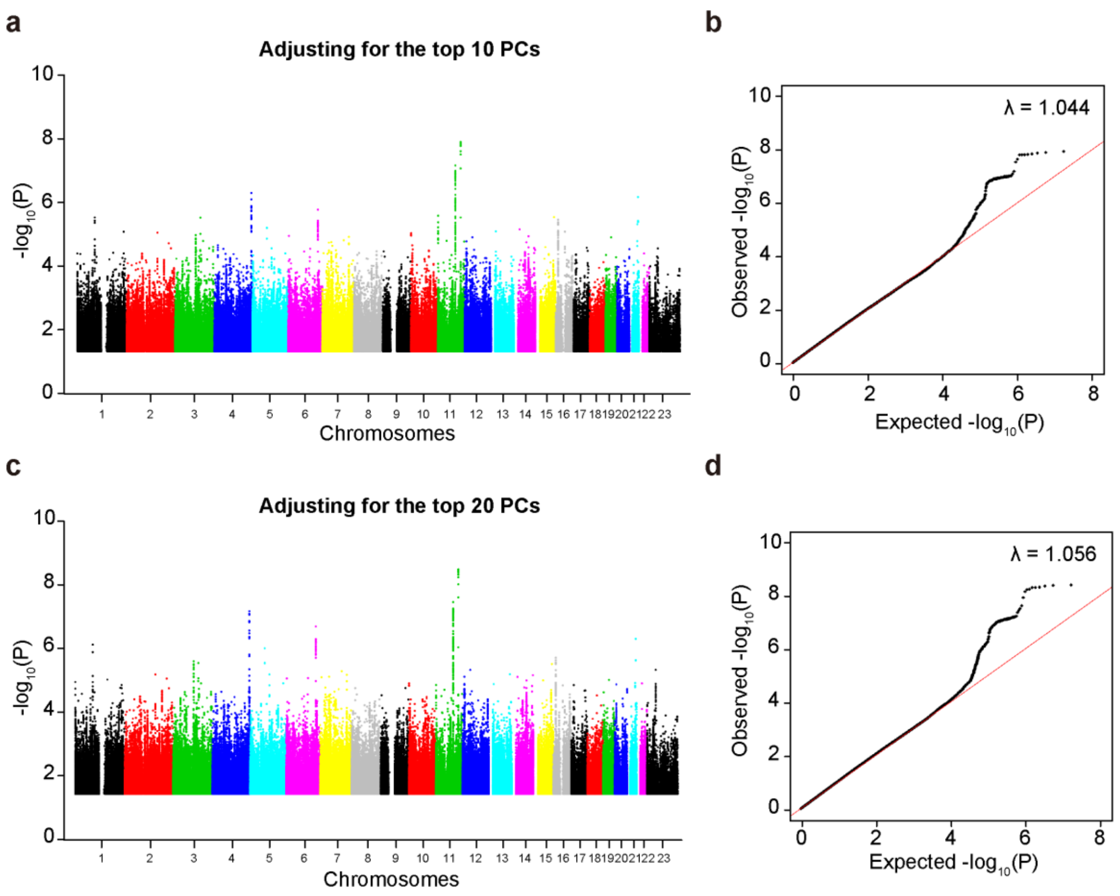

Supplementary Fig. S3 Manhattan plots and quantile-quantile plots for the genome-wide associations in analyses corrected for 10 or 20 PCs. a,c The Manhattan plots showing the genome-wide association statistics for the genotyped and imputed SNPs in the meta-analyses combining the Huoshenshan and Union cohorts, using logistic regression analyses under the additive model, adjusting for age, gender, comorbidities (including hypertension, type 2 diabetes and coronary artery diseases) and the top 10 principal components (**a**) or the top 20 principal components (**c**). Meta-analyses of the associations generated from the Huoshenshan and Union cohorts were conducted using a fixed-effect model. The x-axis represents the genomic position (based on human genome assembly hg19), and the y-axis shows the $-\log_{10}$ (*P*). **b,d** The quantile-quantile plots for the meta-analyses combining the Huoshenshan

and Union cohorts, using logistic regression analyses under the additive model, adjusting for age, gender, comorbidities (including hypertension, type 2 diabetes and coronary artery diseases) and top 10 principal components (**b**) and top 20 principal components (**d**). The red line represents the null hypothesis of no true association. The genomic inflation factor lambda (λ) is defined as the ratio of the medians of the sample χ^2 test statistics and the 1-d.f. χ^2 distribution (0.455).

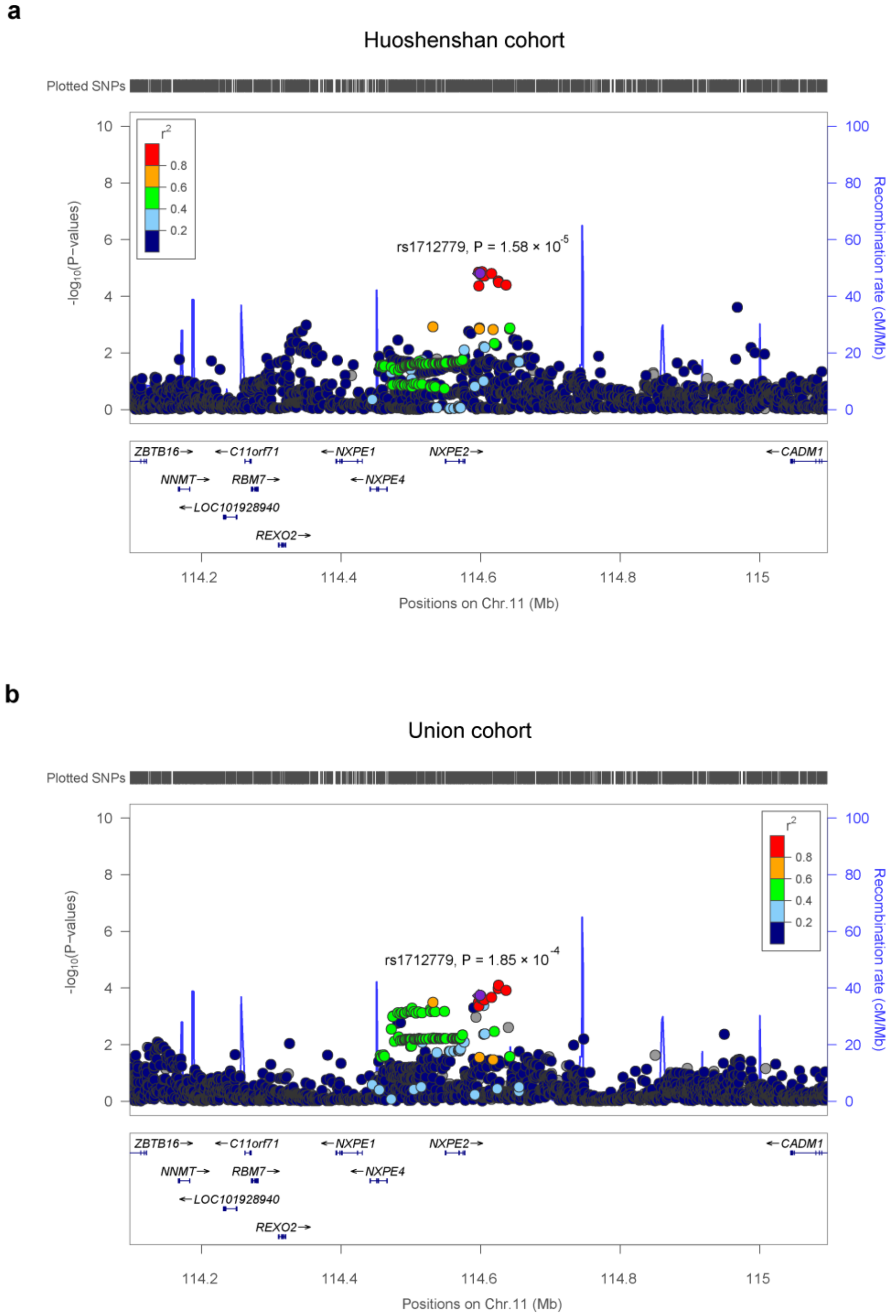
a
Huoshenshan cohort
Plotted SNPs
-log10(P-values)
Recombination rate (cM/Mb)
r2
rs1712779, P = 1.58 × 10-5
ZBTB16
C11orf71
NXPE1
NXPE2
CADM1
NNMT
RBM7
NXPE4
LOC101928940
REXO2
114.2
114.4
114.6
114.8
115
Positions on Chr.11 (Mb)
b
Union cohort
Plotted SNPs
-log10(P-values)
Recombination rate (cM/Mb)
r2
rs1712779, P = 1.85 × 10-4
ZBTB16
C11orf71
NXPE1
NXPE2
CADM1
NNMT
RBM7
NXPE4
LOC101928940
REXO2
114.2
114.4
114.6
114.8
115
Positions on Chr.11 (Mb)

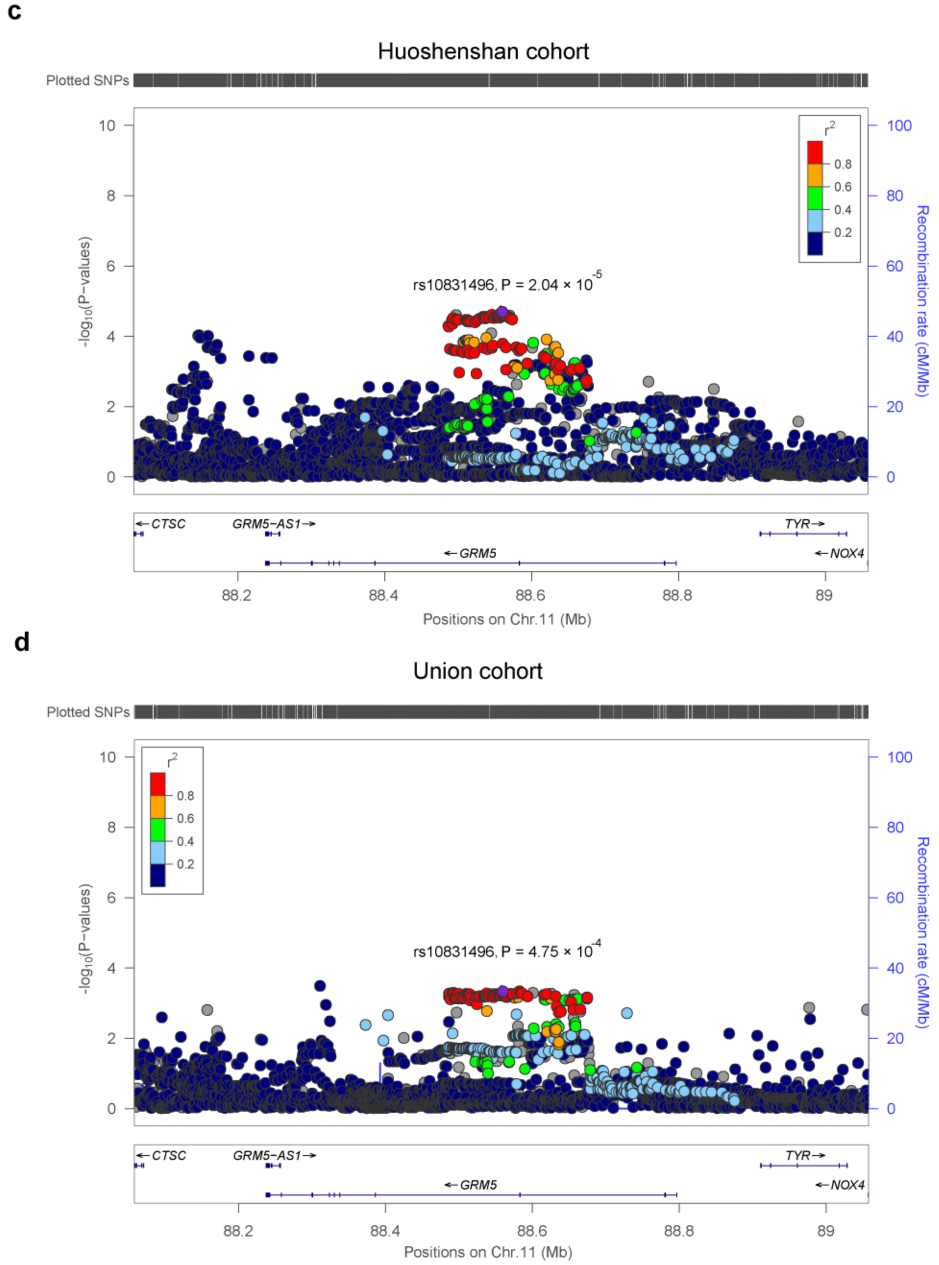

Supplementary Fig. S4 Regional plots for the associations in the main analyses in regions surrounding the rs1712779 and rs10831496, respectively, in the Huoshenshan and Union cohorts, respectively. Results are shown for the SNPs within 1 mega bases (Mb) regions surrounding the index SNP rs1712779 in the Huoshenshan (**a**) and Union cohorts (**c**), respectively, and the index rs10831496 in the Huoshenshan (**b**) and Union cohorts (**d**), respectively. Genomic positions are based on

human genome assembly hg19. The *P* values of rs1712779 and rs10831496 were shown as purple dots. The linkage disequilibrium (LD) values (r^2) to the index SNPs (rs1712779 or rs10831496) for the other SNPs are indicated by marker color. Red signifies $r^2 \geq 0.8$, with orange $0.6 \leq r^2 < 0.8$, green $0.4 \leq r^2 < 0.6$, light blue $0.2 \leq r^2 < 0.4$ and blue $r^2 < 0.2$. The estimated recombination rates from the East Asian populations are derived from the 1,000 Genomes Project (Phase 3, November, 2014), and are plotted in light blue. The East Asian populations were from the 1,000 Genomes Project, consisting of 504 subjects from CHB (Han Chinese in Beijing, China), CHS (Southern Han Chinese), CDX (Chinese Dai in Xishuangbanna, China), JPT (Japanese in Tokyo, Japan) and KHV (Kinh in Ho Chi Minh City, Vietnam). Chr., chromosome. SNP, single nucleotide polymorphism. cM, centi Morgan.

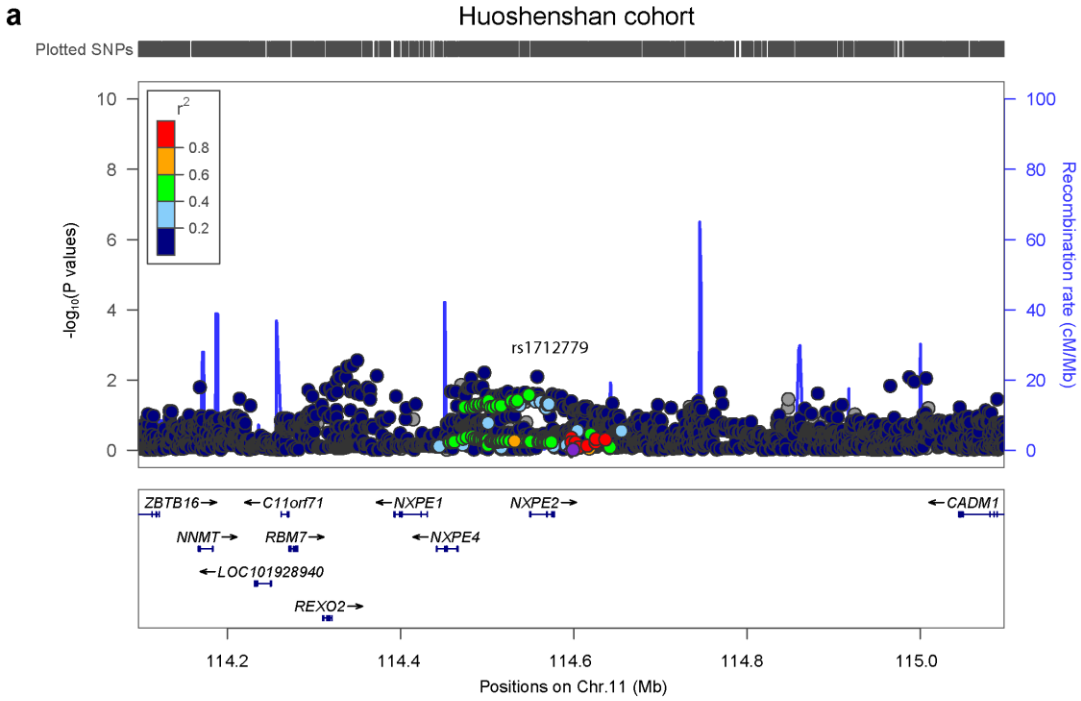
a
Huoshenshan cohort
Plotted SNPs
rs1712779
-log10(P values)
Recombination rate (cM/Mb)
ZBTB16
C11orf71
NXPE1
NXPE2
CADM1
NNMT
RBM7
NXPE4
LOC101928940
REXO2
114.2
114.4
114.6
114.8
115.0
Positions on Chr.11 (Mb)

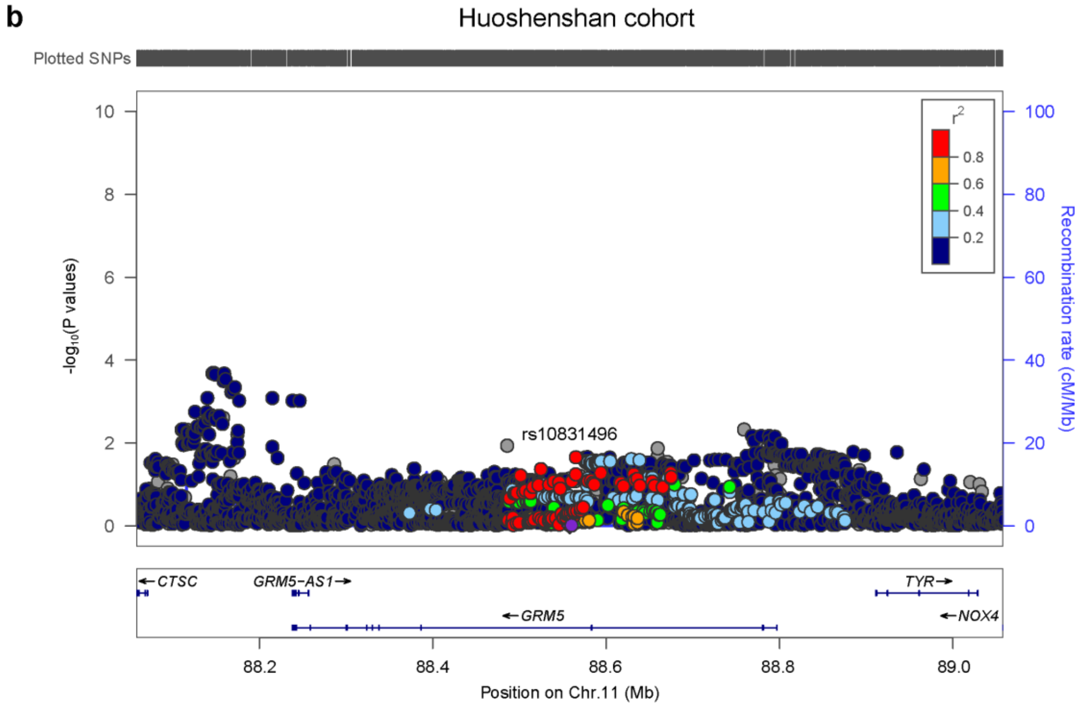
b
Huoshenshan cohort
Plotted SNPs
rs10831496
-log10(P values)
Recombination rate (cM/Mb)
CTSC
GRM5-AS1
TYR
GRM5
NOX4
88.2
88.4
88.6
88.8
89.0
Position on Chr.11 (Mb)

341

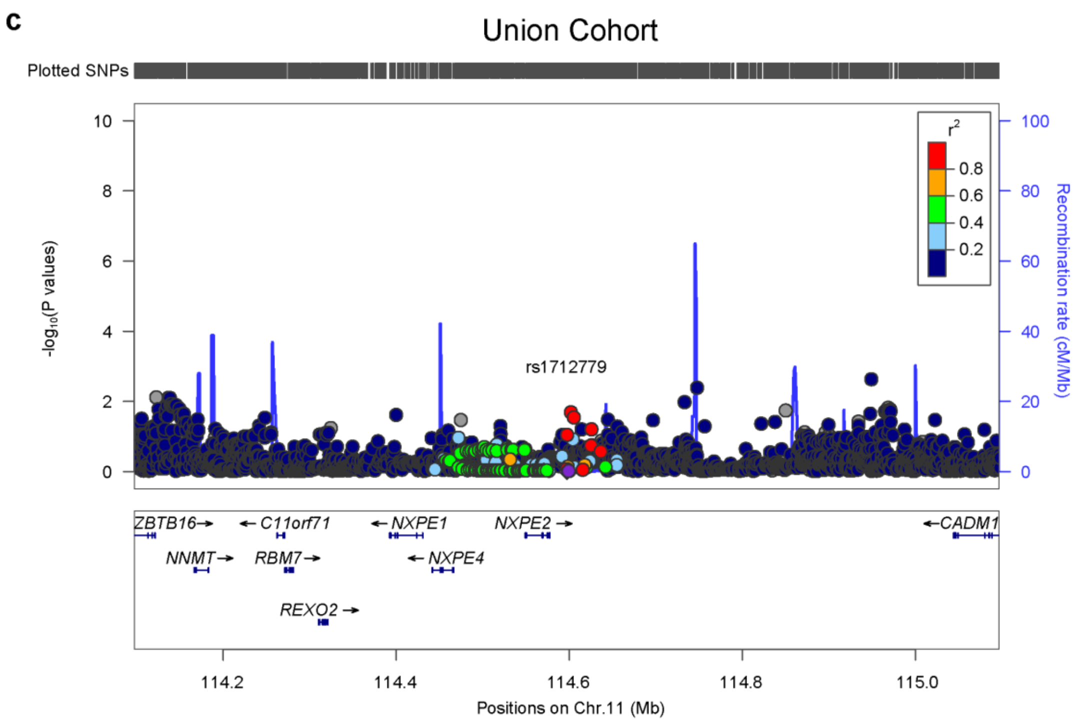
c
Union Cohort
Plotted SNPs
-log10(P values)
Recombination rate (cM/Mb)
r2
rs1712779
ZBTB16
C11orf71
NXPE1
NXPE2
CADM1
NNMT
RBM7
NXPE4
REXO2
114.2
114.4
114.6
114.8
115.0
Positions on Chr.11 (Mb)

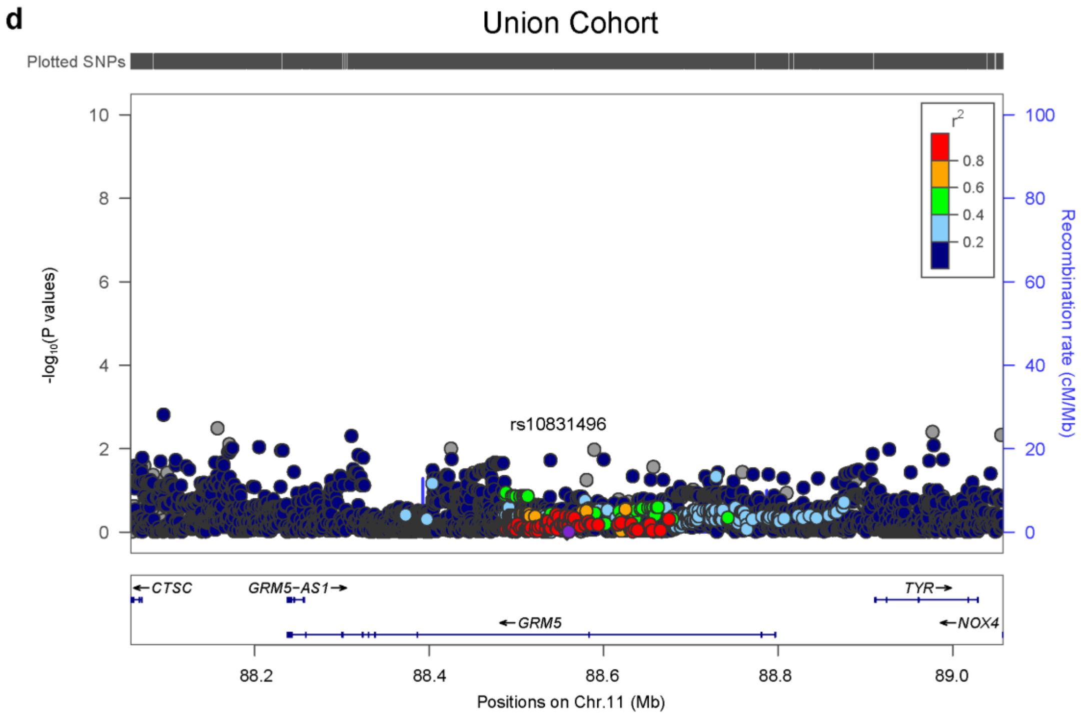
d
Union Cohort
Plotted SNPs
-log10(P values)
Recombination rate (cM/Mb)
r2
rs10831496
CTSC
GRM5-AS1
TYR
GRM5
NOX4
88.2
88.4
88.6
88.8
89.0
Positions on Chr.11 (Mb)

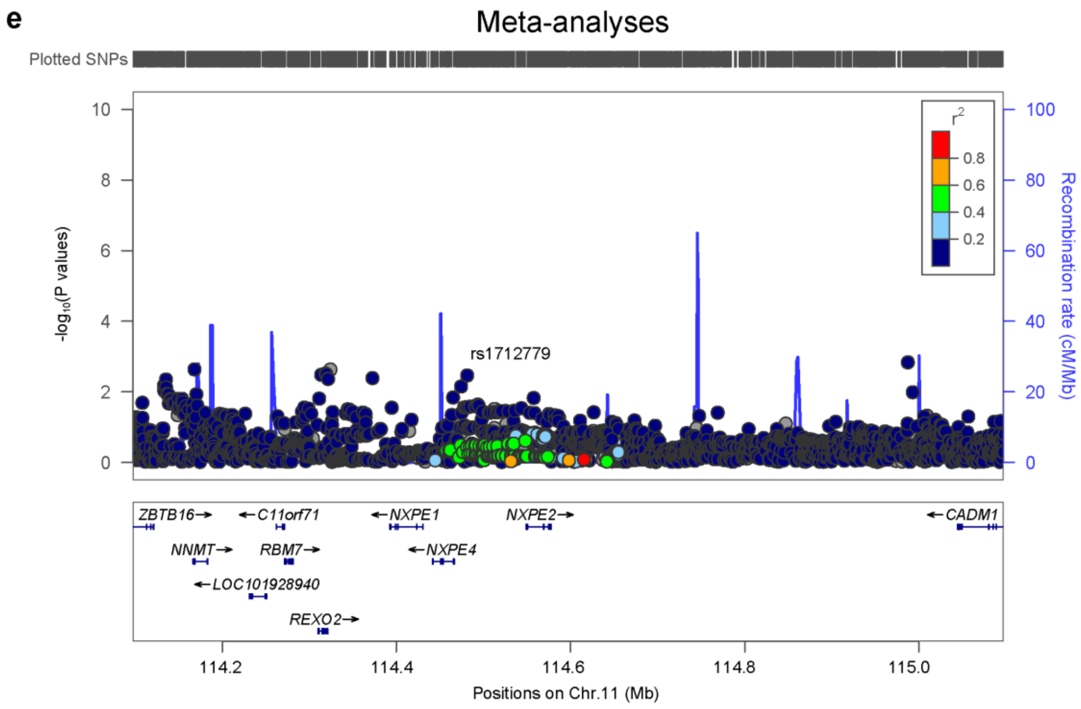

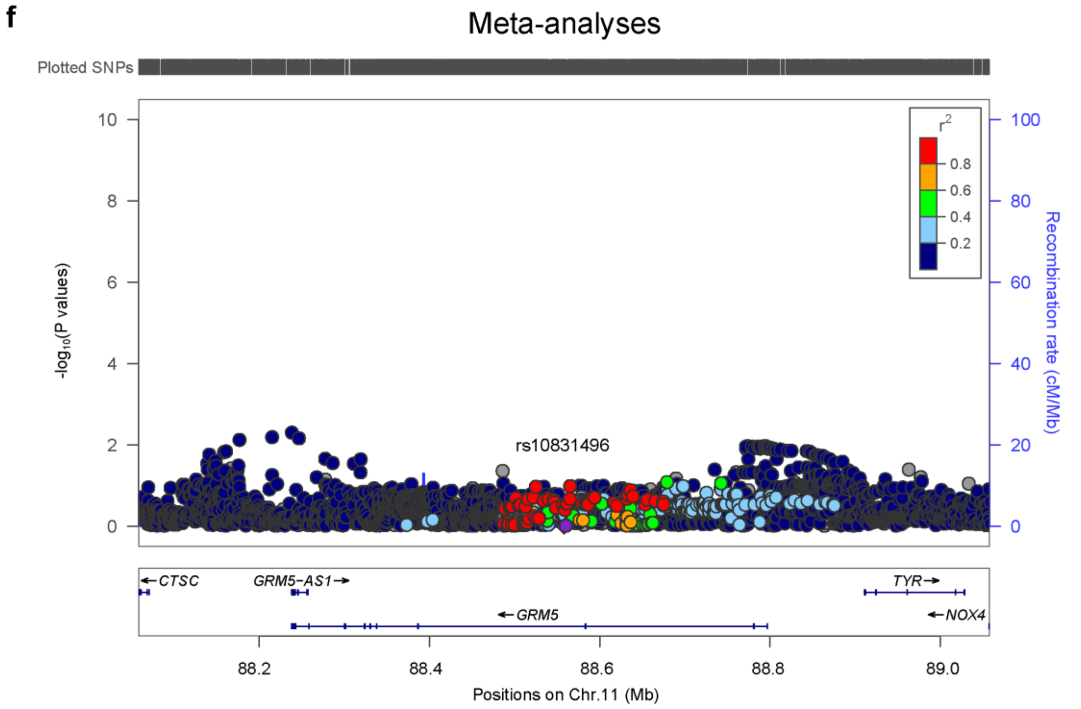

Supplementary Fig. S5 Regional plots for the associations in regions surrounding the rs1712779 or rs10831496 when adjusting for the effect of rs1712779 or rs10831496. We performed association tests for the other SNPs within 1 mega bases (Mb) region surrounding the index rs1712779 on chromosome 11q23.3 in

Huoshenshan cohort (**a**), Union cohort (**c**) and meta-analyses combining these two cohorts (**e**), respectively, adjusting for the effect of rs1712779, as well as age, gender, comorbidities and top five principal components. Similarly, the association tests were performed for the other SNPs surrounding the index rs10831496 on chromosome 11q14.2 in the Huoshenshan cohort (**b**), Union cohort (**d**) and meta-analyses combining these two cohorts (**f**), respectively, adjusting for the effect of rs10831496, as well as age, gender, comorbidities and the top five principal components. No other SNPs on chromosome 11q23.3 or 11q14.2 loci showed evidence of association ($P >$ 0.001). Genomic positions are based on human genome assembly hg19. The linkage disequilibrium (LD) values (r^2) to the index SNPs (rs1712779 or rs10831496) for the other SNPs are indicated by marker color. Red signifies $r^2 \geq 0.8$, with orange $0.6 \leq r^2 < 0.8$, green $0.4 \leq r^2 < 0.6$, light blue $0.2 \leq r^2 < 0.4$ and blue $r^2 < 0.2$. The estimated recombination rates in East Asian populations derived from the 1,000 Genomes Project (Phase 3, November, 2014) are plotted in light blue. The East Asian populations were from the 1,000 Genomes Project, consisting of 504 subjects from CHB (Han Chinese in Beijing, China), CHS (Southern Han Chinese), CDX (Chinese Dai in Xishuangbanna, China), JPT (Japanese in Tokyo, Japan) and KHV (Kinh in Ho Chi Minh City, Vietnam). Chr., chromosome. SNP, single nucleotide polymorphism. cM, centi Morgan.

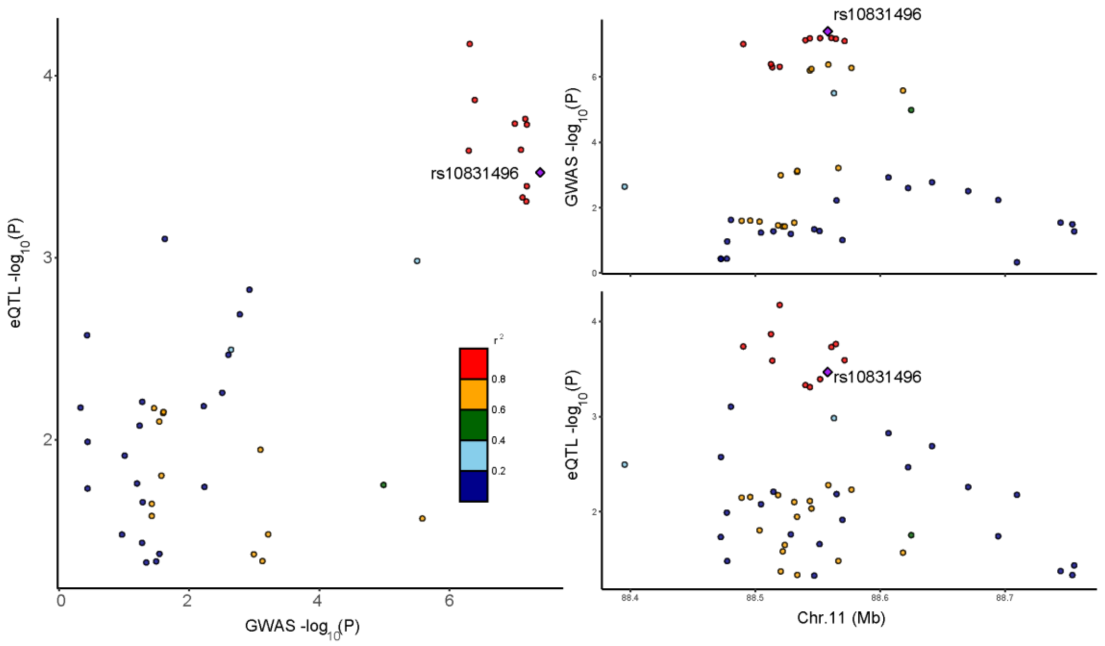

Supplementary Fig. S6 Colocalization analyses of association signals from the eQTLs and GWAS at rs10831496 locus. The GWAS results are from this study. The eQTL results for *CTSC* are from a previous study[4], which consists of 1,799 blood samples from South Asian population. The LD values (r^2) between the index SNP rs10831496 and the other SNPs are based on South Asian populations (from the 1,000 Genomes Project, Phase 3). We performed the colocalization analysis of eQTLs and GWAS associations using the R package coloc (v3.2.1) (Supplementary Methods), and achieved a posterior probability of hypothesis 4 (PP4) score of 0.91, suggesting that the eQTLs and GWAS associations were highly likely to colocalize.

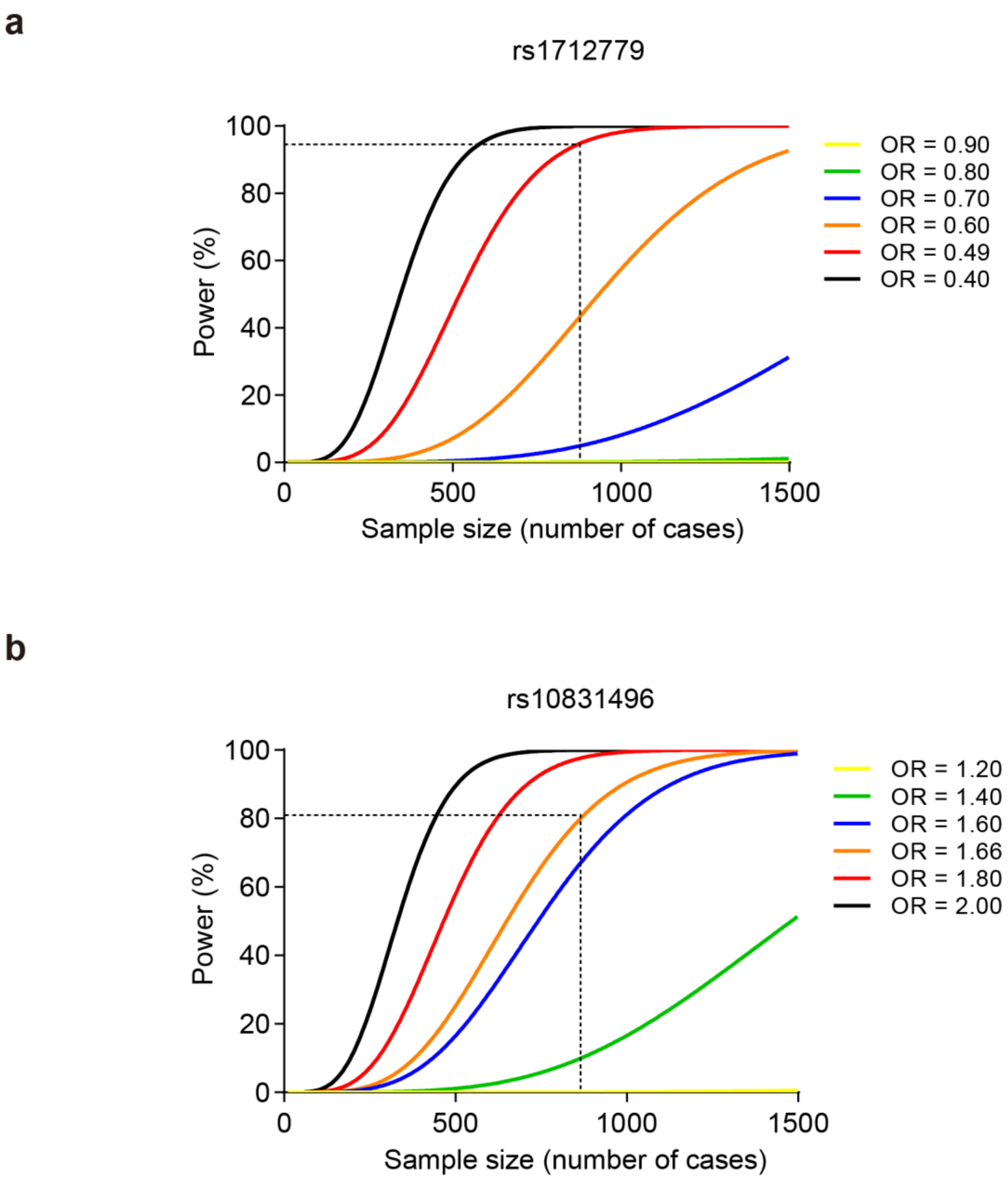

Supplementary Fig. S7 Power to detect the genetic effects of various sizes *versus* sample sizes. Power is reported here as the probability of SNPs to be identified in a scan. Vertical and horizontal dashed lines show that the powers of our GWAS to identify the rs1712779 (**a**) and rs10831496 (**b**) were estimated to be 95% and 81%, respectively, giving the severe/critical COVID-19 prevalence of 15%, 863 cases and 529 controls, P value of 5.0×10^{-8}, an OR of 0.49 and 1.66 for rs1712779 and

rs10831496, respectively, and minor allele frequency of 0.166 and 0.193 for rs1712779 and rs10831496, respectively. OR, odds ratio.

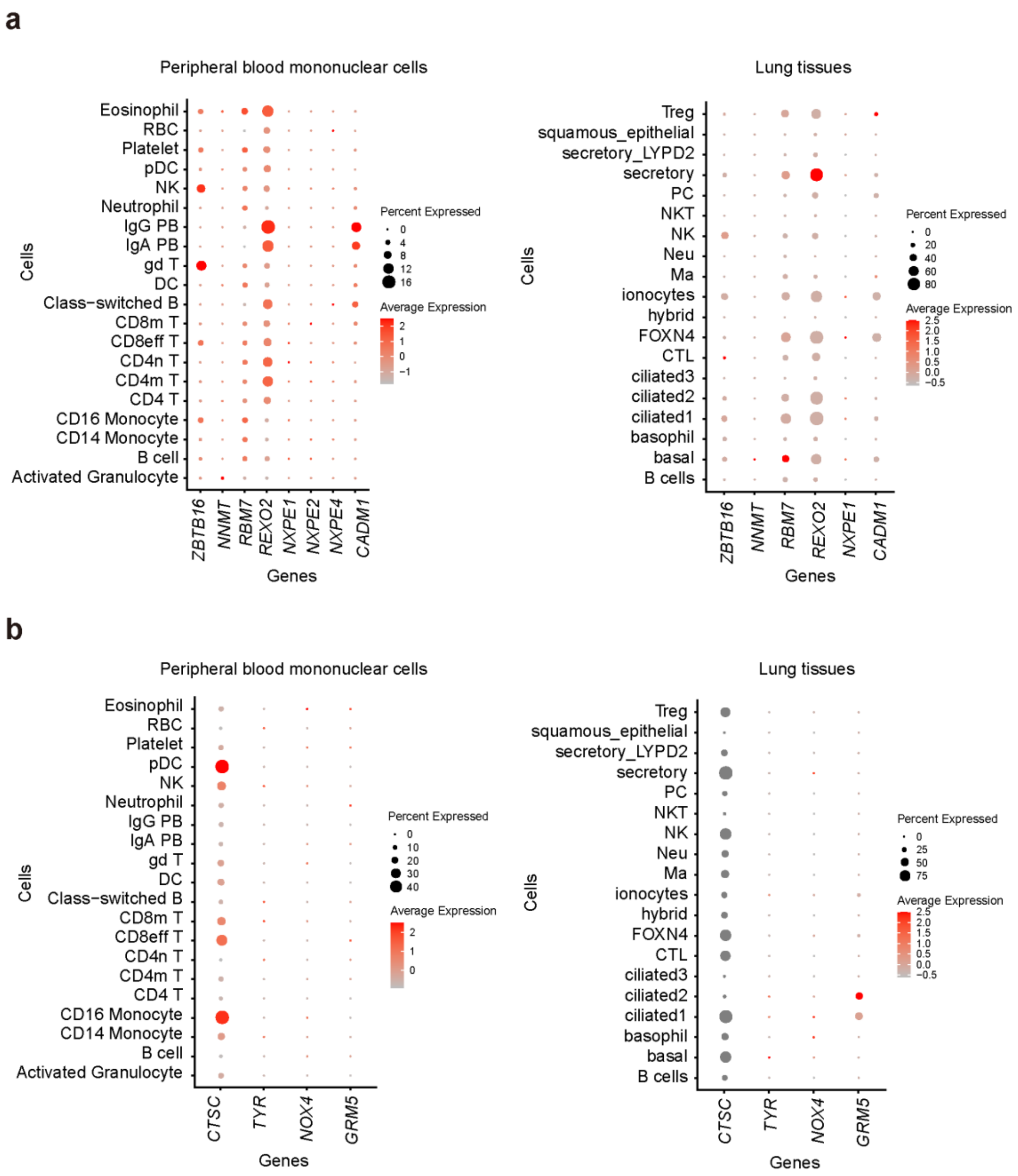

Supplementary Fig. S8 Expression levels of the candidate genes at 11q23.3 and 11q14.2 in peripheral blood mononuclear cells and lung tissues. Two 10x Genomics single cell RNA-sequencing (scRNA-seq) datasets (for peripheral blood mononuclear cells (PBMCs) and lung tissues, respectively) were obtained from two previous studies (PMID: 32514174 and 32591762). Expression levels of the candidate genes at 11q23.3 (**a**) and 11q14.2 (**b**) were shown. The PBMCs dataset consists of 28,094 cells from 8 PBMC samples from 7 COVID-19 patients, and the lung tissues

dataset consists of 38,658 cells from 2 COVID-19 patients. Figures represent mean expressions (visualized by color) of the candidate genes and fraction of cells expressing those genes (visualized by the size of the dot). There are nine genes located at 11q23.3. In the scRNA-seq dataset for PBMCs, the expressions of *C11orf71* gene were not detected, thus only eight genes at 11q23.3 were shown. In the scRNA-seq dataset for lung tissues, the expressions of *C11orf71*, *NXPE4* and *NXPE2* were not detected, thus only six genes at 11q23.3 were shown. There are four genes located at 11q14.2. All the four genes were included in the two scRNA-seq datasets. CD4m T, CD4 memory T cell; CD4n T, CD4 naïve T cell; CD8eff T, CD8 effector T cell; CD8m T, CD8 memory T cell; CTL, cytotoxic T lymphocyte; DC, dendritic cell; FOXN4, FOXN4^{+} cells; gd T, gamma delta (γδ) T cells; IgA PB, IgA peripheral blood B cells; IgG PB, IgG peripheral blood B cells; Ma, macrophage; Neu, neutrophils; NK, natural killer cells; pDC, plasmacytoid dendritic cell; RBC, red blood cell; Treg, regulatory T cells.

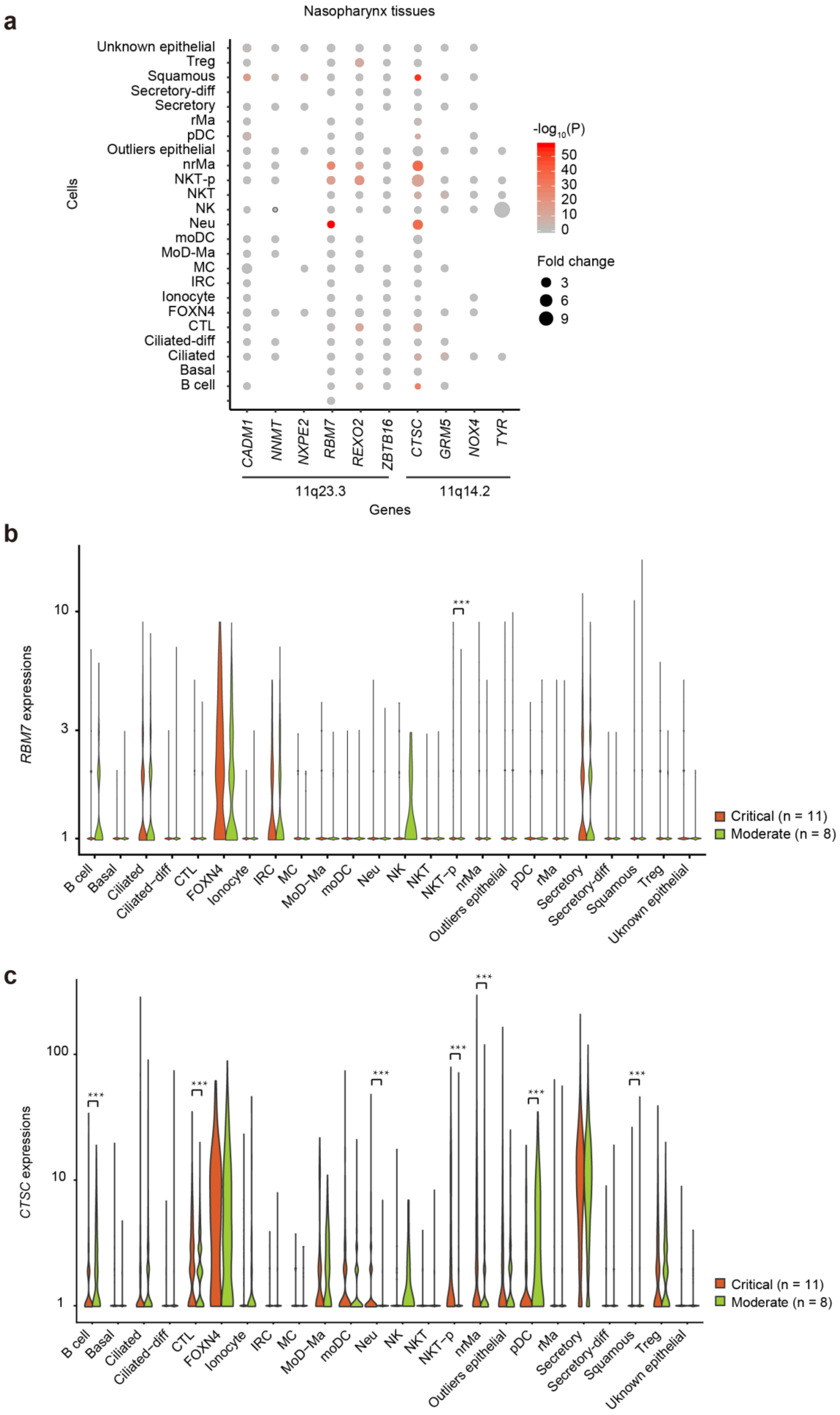
a
Nasopharynx tissues
Cells
Unknown epithelial
Treg
Squamous
Secretory-diff
Secretory
rMa
pDC
Outliers epithelial
nrMa
NKT-p
NKT
NK
Neu
moDC
MoD-Ma
MC
IRC
Ionocyte
FOXN4
CTL
Ciliated-diff
Ciliated
Basal
B cell
CADM1
NNMT
NXPE2
RBM7
REXO2
ZBTB16
CTSC
GRM5
NOX4
TYR
11q23.3
11q14.2
Genes
-log10(P)
50
40
30
20
10
0
Fold change
3
6
9
b
RBM7 expressions
10
3
1
***
Critical (n = 11)
Moderate (n = 8)
B cell
Basal
Ciliated
Ciliated-diff
CTL
FOXN4
Ionocyte
IRC
MC
MoD-Ma
moDC
Neu
NK
NKT
NKT-p
nrMa
Outliers epithelial
pDC
rMa
Secretory
Secretory-diff
Squamous
Treg
Uknown epithelial
c
CTSC expressions
100
10
1
***
Critical (n = 11)
Moderate (n = 8)
B cell
Basal
Ciliated
Ciliated-diff
CTL
FOXN4
Ionocyte
IRC
MC
MoD-Ma
moDC
Neu
NK
NKT
NKT-p
nrMa
Outliers epithelial
pDC
rMa
Secretory
Secretory-diff
Squamous
Treg
Uknown epithelial

Supplementary Fig. S9 Different expression patterns for the candidate genes at 11q23.3 and 11q14.2 in nasopharynx tissues from the critical and moderate COVID-19 patients. a Different expression patterns for the candidate genes at 11q23.3 and 11q14.2 in nasopharynx tissues. The 10x Genomics single cell RNA-sequencing (scRNA-seq) dataset of nasopharynx tissues was obtained from a previous study (PMID: 32591762). This dataset consists of 135,600 cells from 24 donors (including 11 critical COVID-19 patients, 8 moderate COVID-19 patients and 5 healthy individuals). The FIndmarkers function in Seurat (v3.0) was used to identify differentially expressed genes with statistical significance (fold change [FC] $\geq$ 1.5 and adjusted P < 0.05) between the critical and moderate COVID-19 patients. We identified *RBM7*, *REXO2* and *CADM1* at 11q23.3 locus and *CTSC* at 11q14.2 locus as differentially expressed genes. *P* value adjustment was performed using bonferroni correction based on the total number of analyzed genes. Figures represent $-\log_{10}$(adjusted *P* values) (visualized by color) and fold changes (visualized by the size of the dot) for the candidate genes. There are nine and four genes located at 11q23.3 and 11q14.2, respectively. In this scRNA-seq dataset, the expressions of *C11orf71*, *NXPE4* and *NXPE1* at 11q23.3 were not detected, thus only six genes at 11q23.3 and four genes at 11q13.2 were shown. **b**,**c** Expression levels of *RBM7* (**b**) and *CTSC* (**c**) in each cell type of nasopharynx tissues from the critical and moderate COVID-19 patients and healthy subjects. The *RBM7* and *CTSC* mRNA expressions were quantified by 10x Genomics scRNA-seq. The x-axis represents the different types of cells from nasopharynx tissues, and the y-axis shows the $\log_2$

(RNA-sequencing reads count + 1). We observed that the mRNA expression levels of *RBM7* were significantly upregulated in proliferating natural killer T cells (NKT-p) (FC = 1.7 and adjusted $P = 5.82 \times 10^{-15}$). We observed that the mRNA expression levels of *CTSC* were significantly upregulated in cytotoxic T lymphocytes (CTL) (FC = 1.8 and adjusted $P = 2.87 \times 10^{-9}$), neutrophils (Neu) (FC = 3.0 and adjusted $P = 6.44 \times 10^{-38}$), NKT-p (FC = 5.4 and adjusted $P = 1.65 \times 10^{-13}$) and non-resident macrophage (nrMA) (FC = 3.4 and adjusted $P = 6.44 \times 10^{-38}$), while those were significantly downregulated in B cells (FC = 0.3 and adjusted $P = 1.08 \times 10^{-28}$), plasmacytoid dendritic cells (pDC) (FC = 0.2 and adjusted $P = 1.24 \times 10^{-9}$) and squamous cells (FC = 0.5 and adjusted $P = 1.71 \times 10^{-53}$). These results were consistent with the roles of CTSC in modulating the cytotoxicity and inflammatory responses that have been widely investigated (PMID: 25520721 and 33450198). ***, adjusted $P < 0.001$. B cell, B lymphocyte; Basal, basal cell; Ciliated, ciliated cell; Ciliated-diff, differentiated ciliated cell; CTL, cytotoxic T lymphocyte; FOXN4, FOXN4^{+} cell; IRC, IFNG responsive cell; MC, mast cell; MoD-Ma, monocyte-derived macrophage; moDC, monocyte-derived dendritic cell; Neu, neutrophil; NK, natural killer cell; NKT, natural killer T cell; NKT-p, proliferating natural killer T cell; nrMa, non-resident macrophage; pDC, plasmacytoid dendritic cell; rMa, resident macrophage; Secretory, secretory cell; Secretory-diff, differentiated secretory cell; Squamous, squamous cell; Treg, regulatory T cell.

References

1. Li, Y. *et al.* Genome-wide association study identifies 8p21.3 associated with persistent hepatitis B virus infection among Chinese. *Nat Commun* **7**, 11664 (2016).
2. Berisa, T. & Pickrell, J. K. Approximately independent linkage disequilibrium blocks in human populations. *Bioinformatics* **32**, 283-285 (2016).
3. Watanabe, K., Taskesen, E., van Bochoven, A. & Posthuma, D. Functional mapping and annotation of genetic associations with FUMA. *Nat Commun* **8**, 1826 (2017).
4. Pierce, B. L. *et al.* Mediation analysis demonstrates that trans-eQTLs are often explained by cis-mediation: a genome-wide analysis among 1,800 South Asians. *PLoS Genet* **10**, e1004818 (2014).
5. Tanikawa, C. *et al.* A genome-wide association study identifies two susceptibility loci for duodenal ulcer in the Japanese population. *Nat Genet* **44**, 430-434, S431-432 (2012).
6. Severe Covid, G. G. *et al.* Genomewide Association Study of Severe Covid-19 with Respiratory Failure. *N Engl J Med* **383**, 1522-1534 (2020).
7. Wang, F. *et al.* Initial whole-genome sequencing and analysis of the host genetic contribution to COVID-19 severity and susceptibility. *Cell Discov* **6**, 83 (2020).
8. Secolin, R. *et al.* Genetic variability in COVID-19-related genes in the Brazilian population. *Hum Genome Var* **8**, 15 (2021).
9. Pojero, F. *et al.* The Role of Immunogenetics in COVID-19. *Int J Mol Sci* **22**, 2636 (2021).
10. Chua, R. L. *et al.* COVID-19 severity correlates with airway epithelium-immune cell interactions identified by single-cell analysis. *Nat Biotechnol* **38**, 970-979 (2020).
11. Wilk, A. J. *et al.* A single-cell atlas of the peripheral immune response in patients with severe COVID-19. *Nat Med* **26**, 1070-1076 (2020).
